# A microbiome case-control study of recurrent acute otitis media identified potentially protective bacterial genera

**DOI:** 10.1186/s12866-018-1154-3

**Published:** 2018-02-20

**Authors:** Rachael Lappan, Kara Imbrogno, Chisha Sikazwe, Denise Anderson, Danny Mok, Harvey Coates, Shyan Vijayasekaran, Paul Bumbak, Christopher C. Blyth, Sarra E. Jamieson, Christopher S. Peacock

**Affiliations:** 1 The Marshall Centre for Infectious Diseases Research and Training, School of Biomedical Sciences, The University of Western Australia, Perth, WA Australia; 2 Wesfarmers Centre of Vaccines and Infectious Diseases, Telethon Kids Institute, The University of Western Australia, Perth, WA Australia; 3 Telethon Kids Institute, The University of Western Australia, Perth, WA Australia; 4 Department of Microbiology, PathWest, Perth, WA Australia; 5 School of Medicine, The University of Western Australia, Perth, WA Australia; 6 Princess Margaret Hospital for Children, Perth, WA Australia

**Keywords:** Otitis media, Microbiome, 16S rRNA, *Corynebacterium*, *Dolosigranulum*, *Alloiococcus*, *Turicella*, Nasopharynx, Middle ear

## Abstract

**Background:**

Recurrent acute otitis media (rAOM, recurrent ear infection) is a common childhood disease caused by bacteria termed *otopathogens,* for which current treatments have limited effectiveness. Generic probiotic therapies have shown promise, but seem to lack specificity. We hypothesised that healthy children with no history of AOM carry protective commensal bacteria that could be translated into a specific probiotic therapy to break the cycle of re-infection. We characterised the nasopharyngeal microbiome of these children (controls) in comparison to children with rAOM (cases) to identify potentially protective bacteria. As some children with rAOM do not appear to carry any of the known otopathogens, we also hypothesised that characterisation of the middle ear microbiome could identify novel otopathogens, which may also guide the development of more effective therapies.

**Results:**

Middle ear fluids, middle ear rinses and ear canal swabs from the cases and nasopharyngeal swabs from both groups underwent 16S rRNA gene sequencing. The nasopharyngeal microbiomes of cases and controls were distinct. We observed a significantly higher abundance of *Corynebacterium* and *Dolosigranulum* in the nasopharynx of controls. *Alloiococcus, Staphylococcus* and *Turicella* were abundant in the middle ear and ear canal of cases, but were uncommon in the nasopharynx of both groups. *Gemella* and *Neisseria* were characteristic of the case nasopharynx, but were not prevalent in the middle ear.

**Conclusions:**

*Corynebacterium* and *Dolosigranulum* are characteristic of a healthy nasopharyngeal microbiome. *Alloiococcus, Staphylococcus* and *Turicella* are possible novel otopathogens, though their rarity in the nasopharynx and prevalence in the ear canal means that their role as normal aural flora cannot be ruled out. *Gemella* and *Neisseria* are unlikely to be novel otopathogens as they do not appear to colonise the middle ear in children with rAOM.

**Electronic supplementary material:**

The online version of this article (10.1186/s12866-018-1154-3) contains supplementary material, which is available to authorized users.

## Background

Otitis media (OM) refers to a group of inflammatory conditions of the middle ear and is commonly seen in young children. The disease can be divided into two broad categories; acute OM (AOM) and OM with effusion (OME). AOM involves signs of active infection including fever and irritability, and the middle ear contains purulent fluid with a bulging tympanic membrane. OME is characterised by non-purulent effusion and no signs of acute infection. Recurrent AOM (rAOM), defined as 3 or more episodes of AOM within 6 months; or 4 or more in 12 months [1] is also common, with 17% of children experiencing at least 3 episodes before the age of 1 year [2]. Children with rAOM are commonly prescribed repeated courses of antibiotics, and are often referred to an ear, nose and throat (ENT) surgeon for insertion of ventilation tubes (grommets) to prevent rupturing of the tympanic membrane. In Australia, 73% of children under 12 months of age will have experienced OM at least once, with costs to the health care system estimated at $100 to $400 million AUD [3].

Bacterial pathogens that cause AOM are referred to as otopathogens. The three bacterial species widely recognised as otopathogens are *Streptococcus pneumoniae,* non-typeable *Haemophilus influenzae* and *Moraxella catarrhalis*, which are thought to originate from the nasopharynx and are capable of migrating to the middle ear and persisting within biofilms [4]. Children with AOM are commonly colonised with multiple otopathogens [5], and coinfection with respiratory viruses is common [6]. However, a proportion of children with AOM do not appear to be colonised with any of the known otopathogens [5, 7, 8], implying that there may be other bacteria involved in AOM. *Alloiococcus otitidis* and *Turicella otitidis* have been associated with OM, but their role in the pathogenesis of AOM remains unknown [9, 10].

Antibiotic treatment has been shown to be of limited benefit for AOM [11], and all three otopathogens have exhibited resistance to the antibiotics commonly used to treat it [12]. Additionally, one in five children fitted with grommets will require reinsertion in the future [13]. Considering the limited effectiveness of current treatments, there is a need for alternative therapies for rAOM. Probiotic treatment for rAOM is one alternative that has been investigated in several clinical trials. Probiotics are defined as “live microorganisms, which when administered in adequate amounts, confer a health benefit on the host” [14] and act via mechanisms such as competition for nutrients, stimulation of the immune system and direct inhibition with antibacterial molecules [15]. The effect of probiotics on the recurrence of AOM has been variable, with some studies showing a significant improvement [16–21] and others reporting no change [22–27]. The contradictory results of these studies highlight the importance for the development of probiotics containing bacterial strains that are relevant to the upper respiratory tract environment, active against otopathogens and able to colonise the nasopharynx.

A relatively new area of microbiome research involves the identification of commensal bacteria or bacterial products from a ‘healthy’ microbiome for therapeutic use. Therapies identified by this approach have been successful in resolving relapsing *Clostridium difficile* disease in mice [28], resolving *Salmonella enterica* serovar Typhimurium disease in pigs [29] and inhibiting colonisation and biofilm formation of *Staphylococcus aureus* in the nasopharynx of adult humans [30]. These studies demonstrate that the microbiome can be a source for effective probiotic treatments, which may be a single species that acts specifically against the pathogen of interest, or a combination of multiple commensal species.

We had two hypotheses in this study. Firstly, we hypothesised that there are bacterial pathogens involved in rAOM other than the known otopathogens. We sought to identify potential novel otopathogens by characterising for the first time the microbiome of the middle ear in children with rAOM and comparing it to their nasopharyngeal microbiome. Secondly, understanding that exposure to other children via attendance at day care or the presence of multiple children in the home is a major risk factor for rAOM [31, 32], we hypothesised that children exposed to this risk factor but who have not developed rAOM are carrying nasopharyngeal bacteria that provide protection against the disease. We aimed to identify potentially protective commensal bacteria, which may be of use as a specific probiotic therapy for rAOM, by comparing the nasopharyngeal microbiomes of these rAOM-resistant children with those of children with rAOM.

## Methods

### Patient recruitment

Children under the age of 5 years were recruited into either the case (rAOM-prone) or control (rAOM-resistant) group of the Perth Otitis Media Microbiome (biOMe) study in the Perth metropolitan area of Western Australia from December 2013 to December 2015. Cases were undergoing grommet insertion for physician-diagnosed rAOM and were identified as eligible for inclusion by their ENT surgeon. Children undergoing grommet insertion for OME were excluded from recruitment. Middle ear fluid (MEF) from each ear and a nasopharyngeal swab (NPS) were collected at the time of surgery. A saline middle ear rinse (MER) from each ear and one ear canal swab (ECS) were also collected from a subset of patients (56/93). Healthy controls with no history of rAOM were recruited from a community immunisation clinic. Controls were attending day care or had a sibling up to 5 years of age at the time of collection (i.e. were exposed to previously described major risk factors for rAOM [31, 32]). A NPS sample was collected from controls. A questionnaire on demographics, risk factors and recent antibiotic use was completed for all case and control subjects (see Additional file 1). Controls were matched to cases by age (within 3 months if case less than 1 year of age; within 6 months if case between 1 and 2 years of age; within 12 months if case between 2 and 5 years of age) and season (within 2 weeks of collection time; to the previous year if no match found in recruitment year). Subjects were matched by sex where possible. Exclusion criteria for both groups included diagnosis of cleft lip or palate, immune deficiency or genetic syndrome. All specimens and questionnaire data were obtained with informed written consent from a parent or guardian. Recruitment to the study was approved by the Human Research Ethics Committees (HREC) at Princess Margaret Hospital for Children (2013119/EP), St John of God Health Care (#708) and the University of Western Australia (RA/4/1/6839) as well as by all relevant hospital governance committees.

### Sample collection and storage

All specimens obtained from the cases were collected at the time of grommet surgery by the performing surgeon. ECS specimens were taken from one ear prior to myringotomy (incision of the tympanic membrane) with a sterile FLOQswab (Copan) and were placed in 1 ml skim milk tryptone glucose glycerol broth (STGGB, PathWest). Following myringotomy, MEF specimens from each ear were aspirated into a sterile Argyle™ specimen trap (Covidien) with 2 ml of sterile saline used to flush out the tubing. MER specimens were collected from each ear after aspiration of MEF whereby 2 ml of sterile saline was injected into the middle ear, then aspirated into an Argyle™ trap. A NPS was taken with a sterile FLOQswab, rotating for at least 3 s at the nasopharynx before transferral into 1 ml of STGGB. Specimens from the cases were immediately frozen on dry ice or kept on wet ice and transported to the laboratory on the same day. NPS specimens were collected from controls in the same manner and kept on wet ice until transport to the lab. All specimens were frozen at − 80 °C until DNA was extracted.

### DNA extraction and sequencing preparation

Swab samples (NPS and ECS) were first prepared by vortexing followed by transferral of the swab, inverted, to a new sterile tube with sterile forceps. This was centrifuged to collect mucus attached to the swab which was then transferred back into the milk broth. All samples were then aliquoted for DNA extraction (500 μl for ECS and NPS, 750 μl for MEF and MER) and each MEF and NPS specimen was also aliquoted for viral typing (200 μl). The remainder of all samples were archived at − 80 °C. DNA was extracted with the Wizard SV Genomic DNA Purification System (Promega) and FastPrep Lysing Matrix B tubes (MP Biomedicals) as described in Teo et al. [33] with some modifications. In brief, extractions were carried out inside a class II biohazard hood with UV-sterilised plastics and pipettes wiped with DNA Away (Molecular BioProducts) to minimise contamination. A negative extraction control (reagents with no specimen) was included in each extraction batch and each batch included samples of each type. DNA extraction aliquots were then processed as previously described [33], with the final purified genomic DNA stored in DNA Lo-Bind tubes (Eppendorf). Samples were quantified by the Qubit 2.0 fluorometer (dsDNA HS assay, Invitrogen) and diluted to 5 ng/μl with low TE buffer (10 mM Tris-HCl, 0.1 mM EDTA, Fisher Biotec). All genomic DNA was frozen at − 80 °C until sequencing preparation.

### Viral detection

DNA from the viral typing aliquots for all MEF and NPS specimens (cases and controls) was extracted using an automatic extraction platform (MagMAX express 96) according to manufacturer’s instructions. These samples were screened for 19 common respiratory viruses (see Table 1) using a routine multiplex PCR at PathWest (Perth, Western Australia). This method was developed and described by Chidlow et al. [34].

**Table 1:** Gene targets for multiplex respiratory virus PCR. Where multiple strains of a virus were detected, results were combined

Virus name (strains targeted)	Abbreviation	PCR target
Human adenovirus	HAdV	Hexon gene
Human bocavirus	HBoV	VP1 gene
Influenza virus (A/B/C)	IFV	Haemagglutinin & Matrix gene
Respiratory syncytial virus (Type A/Type B)	RSV	Nucleoprotein
Human metapneumovirus	HMPV	Nucleoprotein
Human coronavirus (OC43/229E/HKU1/NL63)	HCoV	Nucleocapsid (OC43, 229E, NL63)
		ORF1a/b (HKU1)
Parainfluenza virus (1/2/3/4)	HPIV	Nucleoprotein
Rhinovirus (A/B/C)^a^	RV	5’UTR

^a^ Rhinovirus typing used primer pairs reported by Lee et al. [35]

### Positive sequencing control generation

A mock community of 16 bacterial species (MOCK) was used as a positive sequencing control (see Table 2). This included a mixture of Gram positive and Gram negative organisms, including some with known prevalence in the upper respiratory tract and some not expected to be found in this environment. Genomic DNA was extracted from glycerol stocks of each species using the Wizard SV Genomic DNA Purification System, with the exception of *N. meningitidis* which was obtained as a heat-killed stock. Each species separately underwent PCR amplification and AMPure XP purification (described in the following section). The PCR products were quantified by Qubit fluorometry, diluted to equal concentrations and pooled at equal volumes to create the positive sequencing control that was included on each sequencing run. The theoretical expected relative abundance is 6.25% for each species. The positive sequencing control was aliquoted before freezing at − 80 °C to minimise freeze/thaw effects on the DNA. Each sequencing run utilised a separate aliquot, in duplicate.

**Table 2:** Species included in the positive sequencing control. All cultures were obtained from the University of Western Australia’s School of Biomedical Sciences culture collection, with the exception of *N. meningitidis* which was kindly provided by A/Prof Charlene Kahler (UWA) and originally described in Stephens et al. [36]

Species	Strain/ATCC/NCTC no.	Gram
*Staphylococcus aureus*	ATCC 29213	Positive
*Staphylococcus epidermidis*	ATCC 14990	Positive
*Streptococcus pneumoniae*	Strain D39	Positive
*Staphylococcus warneri*	ATCC 27836	Positive
*Moraxella catarrhalis*	ATCC 25238	Negative
Non-typeable *Haemophilus influenzae*	Strain 86-028NP	Negative
*Haemophilus haemolyticus*	ATCC 33390	Negative
*Neisseria meningitidis*	Strain M7	Negative
*Corynebacterium jeikeium*	ATCC 43216	Positive
*Propionibacterium acnes*	ATCC 6919	Positive
*Gemella haemolysans* ^a^	NCTC 10244	Positive
*Klebsiella pneumoniae*	NCTC 8172	Negative
*Pseudomonas aeruginosa*	ATCC 15692	Negative
*Streptococcus salivarius*	not available	Positive
*Veillonella parvula*	ATCC 10790	Negative
*Alloiococcus otitidis*	ATCC 51267	Positive

^a^This species may have been mislabelled as it was identified as *Globicatella* by SILVA (v123) taxonomy [37, 38]

### Amplicon sequencing

Samples were prepared for amplicon sequencing following the Illumina protocol for 16S rRNA gene sequencing (Part # 15044223, Rev. B) with modification to ensure sufficient amplification from samples that yielded low amounts of DNA. The recommended primers (forward: 5’CCTACGGGNGGCWGCAG, reverse: 5’GACTACHVGGGTATCTAATCC) target the V3/V4 region of the 16S rRNA gene [39] with Illumina adapters attached to the 5′ end. The expected length of this targeted region is approximately 465 bp including the amplicon primers. The PCR reaction mix contained 9.5 μl of genomic DNA with a final concentration of 300 nM for each amplicon primer and 1× KAPA HiFi HotStart ReadyMix (KAPA Biosystems). A negative extraction control and a no-template negative PCR control were included on each PCR plate. PCR cycling conditions were 95 °C for 3 min, 30 cycles of 95 °C for 30 s, 55 °C for 30 s and 72 °C for 30 s before a final 72 °C for 5 min and holding at 4 °C. All PCR products were purified with Agencourt AMPure XP magnetic beads (Beckman Coulter) as described in the Illumina protocol. The samples and mock community aliquots were then barcoded by Illumina’s dual indexing strategy (Nextera XT Index Kit v2, Sets A and B, Illumina) as described in the Illumina protocol using the default barcode layout from the Illumina Experiment Manager software v1.11.0. Samples were sent to the Australian Genome Research Facility where they underwent further purification and quality checking followed by dilution and equimolar pooling of all samples at 1 ng/μl. The final pool underwent a band excision with the QIAquick Gel Extraction kit (Qiagen) to select the V3/V4 band (approximately 600 bp with adapters and indexes) for sequencing. The pool was sequenced on the Illumina MiSeq with 2 × 300 bp V3 chemistry and a spike-in of 20% PhiX control. A total of 581 samples including all positive and negative controls were sequenced across four separate sequencing runs of 140–149 samples each. Samples of all types were included on each sequencing run.

### Preprocessing of sequence data

A diagrammatic overview of the entire analysis pipeline is provided in Additional file 2. Demultiplexed paired-end reads in FASTQ format were received from the sequencing centre. Overall run quality was observed with FastQC v0.11.3 [40]. The sequence data was processed via the UPARSE pipeline [41] (using the USEARCH v8.1.1861 algorithm [42]) and QIIME v1.9.1 [43]. Paired-end reads were merged with USEARCH with maximum expected errors set to 1 and length restricted to 440–470 bp. Amplicon primer sequences were removed from these high quality reads using a custom script [44] before conversion to FASTA format with QIIME, concatenation of the data from all four sequencing runs and dereplication (collapsing into unique sequences) with USEARCH. Reads that aligned to the human genome (GRCh38_p2) were removed with Deconseq v0.4.3 [45] using identity threshold 94% and coverage threshold 90%. Remaining reads were clustered into OTUs (operational taxonomic units) at a 97% identity threshold with USEARCH to create a high quality chimera-filtered representative set of OTU sequences. The original raw paired-end reads were then merged as before but without expected error filtering. This set of reads was then aligned to the high quality OTU representative set with USEARCH to assign an OTU to each sequence (97% identity threshold) and create an OTU table. From this point, the sequences were processed within QIIME. Taxonomy was assigned with UCLUST v1.2.22q [42] at 90% identity with the SILVA database v123 [37, 38] and then aligned to the SILVA core alignment with PyNAST v1.2.2 [46]. We selected the SILVA database over the QIIME default database (GreenGenes release 13_8 [47]) as *Dolosigranulum* is not present in the 13_8 release, and is misclassified as *Alloiococcus*. The alignment was filtered and a phylogenetic tree generated with FastTree v2.1.3 [48]. OTUs that failed to align and those with an abundance below 0.005% were removed from the OTU table. Samples with low sequencing depth (77 out of 581 total samples; threshold 1499 reads) were also removed from the table. This OTU table was used for all downstream analysis.

Common practice is to then rarefy this data, which involves subsampling to an even number of sequences per sample to render them comparable. However, this can discard a large volume of usable data and reduces statistical power [49]. We have avoided the statistically inadmissible [49] practice of rarefying throughout this study.

### Covariate and diversity analyses

Data from completed demographic questionnaires included day care attendance, duration of breastfeeding, presence of siblings and smokers in the household, relevant clinical diagnoses (i.e. asthma, allergy and chest, heart or kidney problems), previous admission to hospital for infection and use of antibiotics in the previous month. Statistical analysis of covariates (including detection of respiratory viruses) was done in R v3.3.2 [50] using the Wilcoxon rank sum test for continuous data and Pearson’s Chi-squared test for categorical data (unless any values were below 5, then Fisher’s exact test was used). Odds ratios and associated confidence intervals and *p*-values for the viral detection data were calculated in R with the exact2x2 function in the exact2x2 package [51]. All subjects who contributed at least one analysable sample were included in the analysis of covariates.

Summaries of taxonomic relative abundance were generated with QIIME. Correlation of taxa summaries in QIIME was done in either expected mode (all samples against one theoretical expected mock community) or paired mode (pairs of samples) using the Pearson correlation coefficient with a two-sided permutation test (999 permutations) to calculate a non-parametric p-value.

Alpha (within-sample) diversity was measured using Faith’s Phylogenetic Diversity (PD) [52] and inverse Simpson (IS) [53] indices, calculated per sample in QIIME on a cumulative-sum-scale (CSS) normalised and logged OTU table (see differential abundance method for CSS normalisation). PD is a phylogenetic measure that reflects how much of the phylogenetic tree is covered by the OTUs found in the samples. The IS index is a non-phylogenetic measure that takes into account the richness (number of OTUs) and evenness (relative abundance of OTUs) in the sample, with the reciprocal taken so that larger values represent greater diversity. Median values and statistical tests between groups of samples were calculated in R using the Wilcoxon rank sum test for unpaired samples, and Wilcoxon signed rank for paired samples. Alpha diversity boxplots were generated in R with ggplot2 v2.2.0 [54] and gridExtra v2.2.1 [55].

Beta diversity (between-sample diversity) was calculated using the weighted UniFrac metric [56] in QIIME on the raw OTU table. McMurdie and Holmes [49] demonstrated that weighted UniFrac distances are accurate on raw (unrarefied) data. Two-dimensional principal coordinates analysis (PCoA) plots were also generated in QIIME. Procrustes analysis was used to determine the similarity of the PCoA between pairs of samples collected or treated in two different ways. Comparisons were made between raw and rarefied (subsampled) data, MEF and MER sample types from the same ear, and left and right ears from the same child. The analysis calculates an m^2^ value which describes how similar the paired datasets are, with a smaller number indicating the datasets are more similar. The associated non-parametric *p*-value describes the chance of seeing an m^2^ value at least this extreme in 999 permutations using Monte Carlo simulations. A *p*-value of 0 means that no value as extreme as the calculated m^2^ was observed in these permutations. This was carried out within QIIME and plotted with Emperor [57].

### Co-occurrence analyses

To identify positive and negative correlations between individual OTUs in each sample type, we used SparCC [58], developed specifically for quantifying correlations in compositional microbiome data. Correlations between OTUs were determined separately for each sample type (case NPS, control NPS, MEF, MER) with only one sample per child chosen at random if more than one was available. OTUs that were represented by less than 2 reads per sample on average were removed from the raw OTU table before calculating correlations [58]. The default settings were used and one-sided pseudo *p*-values were calculated (taking into account the direction of correlation) using 100 simulated datasets. For the MEF and MER, where one ear per child was selected at random, the analysis was repeated on a second set of samples with the opposite ear. Correlations with a significant (< 0.05) *p*-value in both sets of samples were considered true positives. Values from the first set are reported for correlations that were validated in this way.

### Differential abundance analyses

Differential abundance of OTUs was assessed using the fitZIG function in the R package metagenomeSeq v1.18.0 [59] in R v3.4.1. fitZIG fits a zero-inflated Gaussian mixture model to test for differentially abundant OTUs between groups, and is also designed for use with microbiome data. Five models were fitted to compare the abundance of OTUs between sample types (case/control NPS, MEF/MER, MEF/NPS, MER/NPS and ECS/MEF). In all models, OTUs that were not present in at least 25% of the samples in that model were filtered from the analysis to reduce false positives. The data were normalised using metagenomeSeq’s CSS normalisation [60]. The models were fitted as follows:

#### Model 1 (case NPS/control NPS)

The model included sex (Male/Female), recent antibiotic usage (within the past month; Yes/No) and length of breastfeeding (Never/Under 6 months/6 to 12 months/Over 12 months/Current) to control for these potential confounders. All nasopharyngeal swabs from both groups were included in the analysis except those with missing data for the above covariates (*n* = 4), leaving 98 control NPS and 86 case NPS for comparison.

#### Models 2–5 (MEF/MER, MEF/NPS, MER/NPS, ECS/MEF)

These models included only samples from the cases and were analysed as within-child pairs. Where multiple samples were available (both left and right ear), one was selected at random (sample Set 1). A second set (sample Set 2) containing the sample from the opposite ear was used for validation (i.e. differentially abundant OTUs with an adjusted *p*-value ≤0.05 in only one of the two sets were considered false positives). For the MEF/MER and ECS/MEF comparisons, the pair included samples from the same ear of each child. Subject ID was a covariate in each of the four models. Included in the analysis were 50 pairs of MEF/MER samples, 75 pairs of MEF/NPS samples, 54 pairs of MER/NPS samples and 33 pairs of ECS/MEF samples.

OTUs whose abundance was significantly different between the two groups compared (FDR-adjusted *p*-value ≤0.05) were retained if their median or mean relative abundance was at least 0.35% in one of the two groups compared. We chose this threshold as it discarded known environmental contaminants abundant in the negative controls (e.g. *Delftia, Lysinibacillus*) but retained low-abundance OTUs not found in the negative controls that are known upper respiratory tract colonisers (e.g. *Veillonella*). Heatmaps representing log CSS normalised OTU counts in the compared groups were created with superheat v0.1.0 [61]. Model coefficients (log_2_ fold-changes; logFC) and *p*-values were derived from the MRcoefs function in metagenomeSeq.

## Results

### Study population characteristics

In our cohort, we assessed several environmental and clinical variables that may influence the risk of OM (see Table 3). The most significant difference observed between cases and controls was recent antibiotic usage, which was significantly more common in the cases. The length of breastfeeding and the presence of any other chronic illness were also significantly different between the two groups; the cases were breastfed for a shorter median time (5 months compared to 10.3 months) and had a higher incidence of chronic illness than the controls. Controls were not significantly different to cases in terms of age, season of collection or sex confirming successful matching of controls to cases at recruitment. We did note a higher proportion of males than females with rAOM in the case group.

**Table 3:** Demographic characteristics of children recruited to the study. This table includes all children who contributed at least one sample to any analysis. *P*-values were calculated by Wilcoxon rank-sum test for continuous data, and Pearson’s χ^2^ test for categorical data (unless any values were less than 5, then Fisher’s exact test was used)

	Case (*n* = 93)	Control (*n* = 103)	Missing data	*p*-value
Age	Median 1.9 years (IQR 1.3–2.8 years)	Median 1.6 years (IQR 1.5–3.2 years)	0	0.91
Sex	58 male (62.4%)	53 male (51.5%)	0	0.12
	35 female (37.6%)	50 female (48.5%)		
Aboriginal or Torres strait islander	2 (2.15%)	1 (0.97%)	0	–
Season^a^	Summer: 7 (7.5%)	Summer: 4 (3.9%)	0	0.39
	Autumn: 20 (21.5%)	Autumn: 16 (15.5%)		
	Winter: 45 (48.4%)	Winter: 53 (51.5%)		
	Spring: 21 (22.6%)	Spring: 30 (29.1%)		
Breastfeeding^b^				
Ever breastfed (yes/no)	83 (89.2%)	98 (95.1%)	1 case (1.1%)	0.15
Duration of breastfeeding	Median 5 months (IQR 1.6–10 months)	Median 10.3 months (IQR 6–13 months)	1 control (1.0%)	6.4 × 10^−5^
Currently breastfeeding	7 (7.5%)	14 (13.6%)		0.17
over 12 months	10 (10.8%)	23 (22.3%)		0.00077
6–12 months	31 (33.3%)	45 (43.7%)		
< 6 months (excluding never)	35 (37.6%)	16 (15.5%)		
never	9 (9.7%)	4 (3.9%)		
Day care or school attendance				
Currently attending	74 (79.6%)	80 (77.7%)	1 control (1.0%)	–
Days of day care or school per week	Median 2.5 days (IQR 2–3 days)	Median 2 days (IQR 1.75–3 days)		0.096
no day care or school	19 (20.4%)	22 (21.4%)		0.43
< 2 days/week	13 (14.0%)	20 (19.4%)		
2–3 days/week	52 (55.9%)	46 (44.7%)		
> 3 days/week	9 (9.7%)	14 (13.6%)		
Age at day care/school start^c^	Median 13.5 months (IQR 10 months – 1.9 years)	Median 12 months (IQR 10 months – 1.5 years)	34 controls (33%)	0.94
Siblings of 5 years of age or younger	46 (49.5%)	70 (68.0%)	1 case (1.1%)	–
Smoker in the household	15 (16.1%)	8 (7.77%)	0	0.069
Antibiotic usage in the past month	61 (65.6%)	11 (10.7%)	1 case (1.1%)	1.9 × 10^−15^
			2 controls (1.9%)	
Any chronic illness^d^	18 (19.4%)	10 (9.71%)	14 cases (15.1%)	0.026
			7 controls (6.8%)	
Ever admitted to hospital for infection	17 (18.3%)	11 (10.7%)	0	0.13

^a^ Season was categorised by months

^b^ Median values exclude children who are currently breastfeeding as breastfeeding has not ceased

^c^ The age at which the child started day care or school; whichever they are currently attending

^d^ Any chronic respiratory, cardiovascular or renal illness including asthma and allergies; or other chronic illness identified by the parents

### Bacterial taxa identified across the samples

A total of 31.7 million raw reads were generated across the four sequencing runs. After read pre-processing and OTU picking, the reads were clustered into a total of 123 OTUs. The full taxonomy of these OTUs is provided in Additional file 3, and the results from the positive and negative sequencing controls can be found in Additional file 4. Table 4 shows the aggregated relative abundance of genus-level taxa from all samples excluding those with a low read count (less than 1499 reads) and sequencing controls. *Moraxella, Haemophilus* and *Streptococcus* were abundant in the nasopharynx of the cases and healthy controls, but the control samples contained *Dolosigranulum* and *Corynebacterium* at higher abundance than the cases. The cases additionally contained *Neisseria* (10.9%), *Gemella* (3.2%)*, Porphyromonas* (3.6%), *Alloprevotella* (2.4%) and *Fusobacterium* (2.7%) where these were almost absent in the controls (each below 0.3%). Of the three major otopathogen genera, *Haemophilus* was the most abundant in the middle ear, contributing to 18.5% of the total reads in middle ear fluids and 3.2% of the rinses. *Streptococcus* and *Moraxella* were less common, and were similarly lower in the MER than the MEF. While *Haemophilus* is prevalent in the fluids, overall the ear samples were dominated by *Alloiococcus* and *Staphylococcus*, with *Turicella* also abundant*.* These genera were not abundant in the nasopharynx of cases or controls. The ear canal did not contain any taxon that was not also seen at similar or higher aggregated relative abundance in the rinses or fluids.

**Table 4:** Genus-level community composition by sample type. Relative abundance was calculated for aggregated counts across all samples of each sample type and is summarised at genus level (i.e. aggregates all OTUs with the same genus assignment). Samples below a total read count of 1499 are not included. Genera with an average relative abundance below 1% are grouped as “Other”

Genus level taxonomy	Control NPS (%)	Case NPS (%)	MEF (%)	MER (%)	ECS (%)
*Corynebacterium*	13.30	1.63	1.35	1.34	2.73
*Turicella*	0.03	0.03	6.72	11.72	13.06
*Staphylococcus*	1.42	0.81	9.94	22.23	27.01
*Alloiococcus*	0.17	0.19	49.84	57.04	53.62
*Dolosigranulum*	16.34	1.86	0.05	0.02	0.03
*Streptococcus*	7.05	15.29	3.52	1.20	1.44
*Neisseria*	0.27	10.95	0.19	0.14	0.06
*Haemophilus*	12.43	18.96	18.52	3.18	1.30
*Moraxella*	47.55	30.80	2.17	0.21	0.10
*Pseudomonas*	0.02	0.01	6.34	1.34	0.12
Other (49 other taxa)	1.42	19.48	1.35	1.59	0.54

Species-level identification could not be achieved with the V3/V4 region. Some taxa contain multiple OTUs (which may not be the same species). It is also possible that multiple species have been classified as the same OTU if the sequences are more than 97% identical.

### Diversity within the nasopharynx and middle ear

**Fig. 1:**
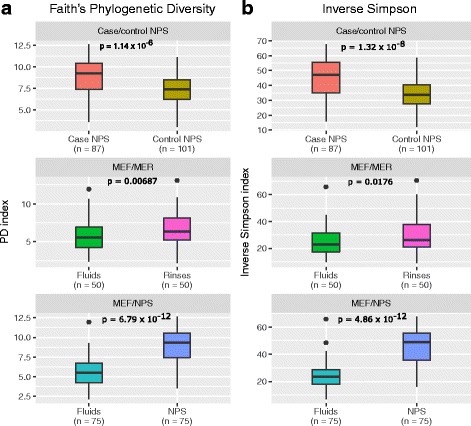
Alpha diversity measured by **a**) Faith’s Phylogenetic Diversity and **b**) Inverse Simpson metrics grouped by sample type. Alpha diversity values were calculated on CSS normalised and logged read counts. The *p*-values represent the difference between groups determined by Wilcoxon rank sum (case/control NPS) or Wilcoxon signed rank (MEF/MER and MEF/NPS) test where paired samples were from the same child

We measured alpha (within-sample) diversity with the Faith’s phylogenetic diversity (PD) and inverse Simpson (IS, alternatively reciprocal Simpson) metrics. Alpha diversity by sample type is shown in Fig. 1. The nasopharynx of children with rAOM was significantly more diverse than the nasopharynx of the healthy controls. Within the same ear of the same child, the middle ear rinse was also significantly more diverse than the fluid but this difference was not as pronounced. The nasopharynx was also more diverse than the middle ear fluid when comparing within the same child.

### Comparing the microbiome of the nasopharynx between rAOM-prone and rAOM-resistant children

Beta diversity represents the differences between samples; how similar or dissimilar they are to each other. We calculated beta diversity with the weighted UniFrac metric [56] on the raw read counts to determine whether there was a distinct microbiome related to case/control status, sample type or other covariates. Calculating beta diversity on raw counts is accurate [49] and was very similar to using rarefied counts (Procrustes analysis, see Additional file 5).

**Fig. 2:**
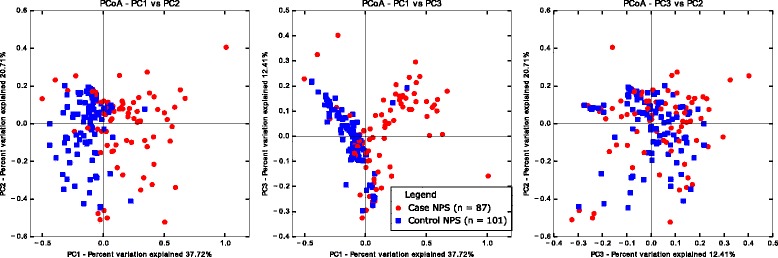
Principal coordinates analysis (PCoA) on nasopharyngeal samples from cases and controls. Distances between samples were calculated using the weighted UniFrac metric [56]

We compared the microbiome of the nasopharynx of cases to that of controls to identify genera that may be associated with rAOM (potential novel pathogens), or with apparent resistance to rAOM (potential candidates for probiotic therapy). Figure 2 shows a principal coordinates analysis (PCoA) plot of the UniFrac distances on the nasopharyngeal samples from cases and controls, where each sample is an individual point. The closer two points are, the more similar the microbiome of those samples. The nasopharyngeal microbiome of the cases was distinct from that of the controls, separating along the PC1 axis; indicating that much of the variability between samples is explained by case/control status. No grouping of samples was observed with any other covariate (including age, presence of chronic illness, attendance at day care or school, sex, previous hospital admission for infection, season of collection and presence of siblings), with the exception of those previously shown to differ significantly between cases and controls. The pattern of recent antibiotic usage overlapped with case/control status, which is expected, as most of the cases had recently taken antibiotics whilst usage in the controls was low. Current breastfeeding and breastfeeding for at least 12 months were also concentrated towards the controls along PC1, who had a higher median length of breastfeeding (see Table 3). There were no batch effects by sequencing run (see Additional file 6).

**Fig. 3:**
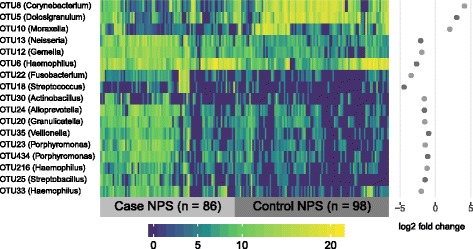
Log CSS normalised counts of differentially abundant OTUs between rAOM-prone and rAOM-resistant children. OTUs shown are significantly differentially abundant between the nasopharyngeal samples of the cases and controls and are additionally found above the threshold of ≥0.35% mean or median relative abundance in at least one group. Differential abundance analysis controlled for recent antibiotic usage, length of breastfeeding and sex; children with missing data for any of these covariates were excluded (*n* = 4). log_2_FC refers to the log fold change of OTU abundance from cases to controls

We then determined which OTUs were causing this difference between the two groups by fitting a model using the fitZIG function in metagenomeSeq [59]. The model controlled for recent antibiotic usage, sex and length of breastfeeding. Of the 33 significantly differentially abundant OTUs, 16 were above a threshold of ≥0.35% mean or median relative abundance in either group (see Methods) and are shown in Fig. 3. Additional file 7 contains a full list of significant OTUs with adjusted *p*-values and log_2_fold-changes between groups for all fitZIG models. Of these 16 OTUs, 14 were more abundant in the nasopharynx of the cases. *Haemophilus* (OTU6) was one of these with a logFC of 2.7 (*p* = 0.004), though it was commonly seen across both groups. The other otopathogen genera *Moraxella* (OTU2) and *Streptococcus* (OTU4) were not significantly different. The remaining 13 OTUs were found at low abundance in the cases, but were very low or almost absent in the controls (logFC = 0.96 to 4.4, *p*-values < 0.01). Those with the highest median log CSS counts were *Gemella* (OTU12) and *Neisseria* (OTU13)*.* The representative sequences for these OTUs did not match any species definitively with BLASTN 2.6.1 [62] against 16S rRNA sequences; OTU12 matched *G. taiwanensis* and *G. haemolysans* at 100%, with OTU13 hitting several *Neisseria* species at 98–99% identity. However, the SILVA database classified the sequence as *N. lactamica*.

Three OTUs were more abundant in the nasopharynx of controls compared to the cases. *Moraxella* (OTU10, distinct from OTU2 which was abundant across all nasopharyngeal samples and was not significantly different here) was found at low relative abundance in both groups, but was prevalent in several controls (logFC = 2.0, *p* = 0.05). The representative OTU sequence for this *Moraxella* OTU matches *M. lincolnii* using BLASTN [62]. *Dolosigranulum* (logFC = 3.0, *p* = 0.002) and *Corynebacterium* (logFC = 4.1, *p* = 8.9 × 10^− 6^) were both low in the cases but significantly more abundant in the controls. *Dolosigranulum* only has one species (*D. pigrum*), but this *Corynebacterium* OTU (OTU8) could not be identified at species level with BLASTN [62].

### Comparing the microbiome of different sample types within children with rAOM

**Fig. 4:**
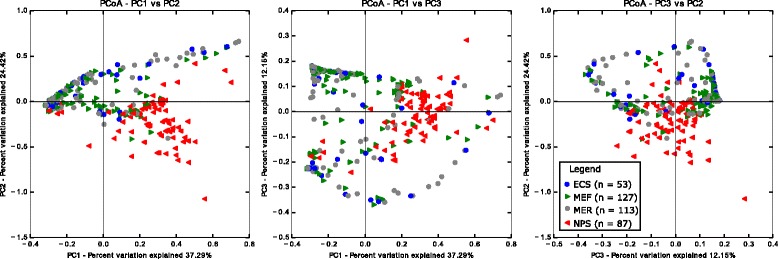
Principal coordinates analysis (PCoA) on samples from children with rAOM. Distances between samples were calculated using the weighted UniFrac metric [56]

We compared the microbiome of the nasopharynx, middle ear and ear canal in children with rAOM to identify novel bacteria that may be involved in the disease. The NPS are distinct from the ear samples in a weighted UniFrac PCoA plot (Fig. 4). The MEF, MER and ECS samples do not form distinct groups and are more sparsely distributed than the NPS. The ear samples (MEF, MER and ECS) were not observed to group by any other covariates (including antibiotic use, age, duration of breastfeeding, presence of other chronic illness, current attendance at day care or school, sex, previous hospital admission for infection, season of collection and presence of siblings), and there were no batch effects by sequencing run (see Additional file 8); suggesting that the sparse layout of these samples in Figure 4 is most likely due to large differences between individual children.

Pairs of left and right ear samples from the same child were strongly correlated. When comparing pairs of taxa summaries, the fluids had a Pearson correlation coefficient of 0.833 (95% CI 0.821–0.844, non-parametric *p* = 0.001, 999 permutations) and the rinses, 0.858 (95% CI 0.849–0.867, non-parametric *p* = 0.001, 999 permutations). They are also moderately similar following Procrustes analysis (see Additional file 9; m^2^ = 0.340, *p* < 0.001, 999 permutations) but many pairs are distant. A lower m^2^ value indicates higher similarity between datasets. To ensure robust results, downstream analyses that required independent samples (i.e. only one ear per child where two were available) were run with the left or right ear randomly chosen (Set 1), and then repeated with the sample from the opposite ear (Set 2) for agreement.

#### Middle ear fluid and middle ear rinse

**Fig. 5:**
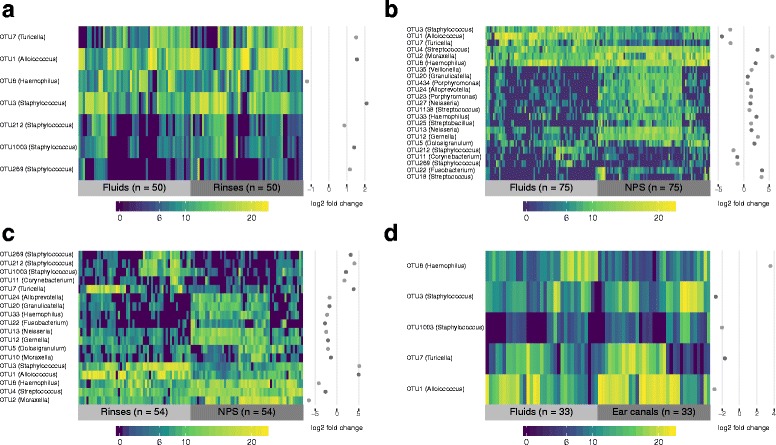
Log CSS normalised counts of differentially abundant OTUs amongst sample types within the cases. OTUs plotted are significantly differentially abundant between paired (within-child) **a**) MEF and MER samples; **b**) MEF and NPS samples; **c**) MER and NPS samples; **d**) MEF and ECS samples. Only OTUs with an adjusted *p* ≤ 0.05 and above the threshold of ≥0.35% mean or median relative abundance in at least one of the groups in each comparison are shown. log_2_FC refers to the log fold change between the two groups, with the value representing the change from **a**) fluids to rinses; **b**) fluids to NPS; **c**) NPS to rinses; **d**) ear canals to fluids

Sampling of the MEF in a subset (60%) of the cases was followed by a saline MER to attempt to capture bacteria that may not otherwise be detected in the fluid (for example, bacteria present in biofilm adhered to the mucosa). The taxonomic composition of the two sample types were strongly correlated when comparing samples from the same ear (Pearson correlation coefficient 0.835, 95% CI 0.826–0.843, non-parametric *p* = 0.001, 999 permutations) and they occupy a similar area on the PCoA plot (Figure 4). Procrustes analysis on the first three principal coordinates between these pairs indicated that the sample types are only moderately similar (m^2^ = 0.335, *p* < 0.001, 999 permutations; see Additional file 10). To determine which OTUs were differentially abundant between the MEF and MER, we fitted a fitZIG model on MEF/MER pairs from the same ear with the Set 1/Set 2 approach (see Methods). The 7 significantly differentially abundant OTUs above threshold are shown in Fig. 5a (two additional OTUs above threshold were dropped for disagreement between Set 1 and Set 2). *Staphylococcus* (OTUs 3, 212, 269 and 1003)*, Alloiococcus* (OTU1) and *Turicella* (OTU7) were found at higher abundance in the MER than in the MEF (logFC = 0.9 to 2.1, *p*-values < 0.0007). *Haemophilus* (OTU6) was more abundant in the MEF than in the MER (logFC = 1.2, *p* = 2.5 × 10^− 9^). While three *Staphylococcus* OTUs (OTU212, OTU269 and OTU1003) were found at very low abundance, all other OTUs were found at moderate abundance in both sites.

#### Middle ear and nasopharynx

We found that the middle ear and nasopharynx of children with rAOM were not highly concordant. In Fig. 4, the NPS samples are distinct from the ear samples (including MEF, MER and ECS). Differential abundance analysis revealed 23 significant OTUs above threshold between the MEF and NPS (an additional 2 OTUs above threshold were dropped for disagreement between Set 1 and Set 2), and 18 OTUs when comparing the MER to the NPS (4 OTUs above threshold dropped for disagreement). The abundance of significant OTUs are shown in Fig. 5b and c. Overall, the genera *Staphylococcus* (OTU3)*, Alloiococcus* (OTU1) and *Turicella* (OTU7) were highly abundant in the middle ear, with very low abundance in the nasopharynx (logFC = 2.6 to 5.2, *p*-values < 0.001); these genera appear to be characteristic of the middle ear. OTU3 matched several species of *Staphylococcus* above 97% with BLASTN, however *Alloiococcus* and *Turicella* both contain one known species each (*A. otitidis* and *T. otitidis* respectively). The otopathogen genera *Haemophilus* (OTU6), *Moraxella* (OTU2) and *Streptococcus* (OTU4) were moderately abundant in both sites but were higher in the nasopharynx (logFC = 2.3 to 6.4, *p*-values < 0.002). Several OTUs including *Gemella* (OTU12) and *Neisseria* (OTU13) were at very low abundance in the middle ear and higher in the nasopharynx, though still low overall (logFC = 0.8 to 3.6, *p*-values < 0.05). These low-abundance OTUs appear to be contributing to the increased diversity seen in the nasopharynx compared to the middle ear. Differences between the middle ear and nasopharynx were generally more pronounced when comparing to the MER samples.

#### Middle ear fluid and ear canal

The ear canal samples were taken to assess which bacteria may potentially contaminate the MEF and MER samples during collection. No bacterial taxa were present in the ear canal that were not present in the middle ear, and the dominant bacteria in the canal were also dominant in the middle ear (see Table 4). Shown in Fig. 5 d, only three OTUs were significantly differentially abundant between pairs of ECS and MEF samples. *Alloiococcus* (OTU1), *Turicella* (OTU7) and *Staphylococcus* (OTU3) occurred at higher abundance in the ear canal (logFC = 2.9, 1.7 and 2.7 respectively; *p*-values < 0.004), though they were common in both sites. *Haemophilus* (OTU6) was higher in the middle ear fluid with a log fold change of 3.6 (*p* = 2.5 × 10^− 13^).

### Patterns of bacterial co-occurrence

**Fig. 6:**
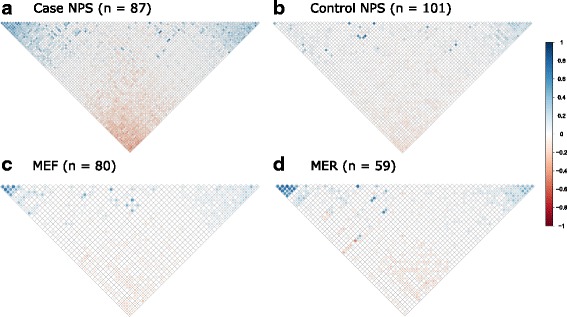
Correlations between OTUs. Correlation coefficients between OTUs were calculated by SparCC [58] within the **a**) Case NPS; **b**) Control NPS; **c**) MEF and **d**) MER. One sample per child was included in each set. Non-significant correlations (one-sided *p* > 0.05) are coloured white. N refers to the number of samples included in each correlation analysis, which tested for correlations between all OTUs observed in those samples

We searched for strong correlations between the dominant OTUs representing the known otopathogen genera (*Haemophilus, Streptococcus* and *Moraxella*) and also between the OTUs we found to be characteristic of the case or control nasopharynx, or the middle ear. Correlations were determined separately for each sample type with SparCC [58]. Correlograms showing the overall pattern of correlations for each sample type are shown in Fig. 6, and a full list of correlations can be found in Additional file 11.

Within the case nasopharynx, there were several moderately strong (0.6–0.8) correlations with *p* < 0.01 (100 simulations). The strongest correlations observed were between low-abundance OTUs *Haemophilus* (OTU1033) and *Moraxella* (OTU1073) with coefficient 0.80; between the most abundant *Moraxella* OTU (OTU2) and *Haemophilus* (OTU1033) with coefficient 0.77 and between *Gemella* (OTU12) and *Porphyromonas* (OTU23) with coefficient 0.70. *Gemella* (OTU12) and *Neisseria* (OTU13), OTUs that were significantly more prevalent in the cases than in controls, were also positively correlated (0.65). Correlations between the dominant OTUs for *Haemophilus* (OTU6), *Moraxella* (OTU2) and *Streptococcus* (OTU4) were not significant.

Within the control NPS samples, there were fewer significant correlations observed. A moderately strong correlation (0.722, *p* < 0.01, 100 simulations) was detected between *Corynebacterium* (OTU5) and *Dolosigranulum* (OTU8), which were significantly more prevalent in the nasopharynx of controls than cases. *Moraxella* (OTU10) correlated only weakly with other OTUs.

In the middle ear, the dominant *Staphylococcus* (OTU3) correlated with other low-abundance *Staphylococcus* OTUs (1003 and 212), with coefficients 0.61 to 0.73 (*p*-values < 0.01, 100 simulations) in both the MEF and MER. These minor *Staphylococcus* OTUs were also moderately to strongly correlated with each other (coefficient 0.83 in MER, 0.71 in MEF). A moderately negative correlation was observed between *Staphylococcus* (OTU3) and *Alloiococcus* (OTU1) in the MER (coefficient = − 0.52, *p* < 0.01, 100 simulations), although this association was weak in the MEF (coefficient = − 0.3, *p* < 0.01, 100 simulations). The genera that appear to be characteristic of the middle ear, *Alloiococcus* (OTU1) and *Turicella* (OTU7), only weakly correlated with each other in the MEF (coefficient = 0.37, *p* < 0.01, 100 simulations) but not in the MER. No significant correlations were found between *Haemophilus* (OTU6), *Streptococcus* (OTU4) and *Moraxella* (OTU2) in either the MEF or MER.

### Detection of respiratory viruses

We tested all MEF and NPS samples for common respiratory viruses. The identification rates (the percentage of positive samples out of the total number of samples tested) are shown in Table 5. Amongst the NPS samples, 61.3% of the cases were positive for at least one virus compared to 47.6% of the healthy controls, though the odds of detecting any virus were not significantly higher in the cases (OR 1.4, (0.8, 2.6)). The odds of detecting respiratory syncytial virus (RSV) were 9.6 times higher (2.2, 60.5) in the cases than the controls. HMPV was also substantially different between the groups; while the detection rate was low in the cases (8.6%) the virus was not detected in any of the controls. For all other viruses tested, the odds of detection were also significantly higher in the cases than in the controls. RV was the most frequently detected virus overall, seen in 43% of case NPS and 34% of control NPS. No samples in either the cases or controls tested positive for HBoV.

**Table 5:** Viral identification rates in the nasopharynx of cases and controls. The identification rate is the percentage of positive samples out of the total number of samples collected for that group

Virus ^a^	Case NPS (*n* = 93)	Control NPS (*n* = 103)	Odds ratio (95% CI)	*P*-value
IFV	1 (1.1%)	2 (1.9%)	–	–
HAdV	18 (19.4%)	6 (5.8%)	3.9 (1.5, 10.3)	0.004
HBoV	0 (0%)	0 (0%)	–	–
RSV	15 (16.1%)	2 (1.9%)	9.6 (2.2, 60.5)	0.0005
HCoV	14 (15.1%)	5 (4.9%)	3.5 (1.1, 10.4)	0.03
HPIV	4 (4.3%)	4 (3.9%)	1.1 (0.3, 4.8)	1
HMPV	8 (8.6%)	0 (0%)	–	–
RV	40 (43.0%)	35 (34.0%)	1.5 (0.8, 2.6)	0.2
Total	57 (61.3%)	54 (47.6%)	1.4 (0.8, 2.6)	0.2

^a^ Viral abbreviations: *IFV* Influenza virus, *HAdV* Adenovirus, *HBoV* Human bocavirus, *RSV* Respiratory syncytial virus, *HCoV* Human coronavirus, *HPIV* Parainfluenza virus, *HMPV* Human metapneumovirus, *RV* Rhinovirus

We calculated the rate of concordance in viral detection between the MEF and NPS for each virus to determine whether any of these viruses are found at both sites in the same child (Table 6). Overall, the concordance rates were low; RV showed the highest concordance rate with 44.4% of cases with RV having detectable virus in both sites. Each virus was detected more frequently in the nasopharynx than in the middle ear, with the exception of IFV which was only detected in one NPS and one MEF from different children.

**Table 6:** Viral concordance rates between the middle ear and nasopharynx of cases. The concordance rate is the number of cases where the virus was identified in both the MEF and NPS out of the total number of cases where the virus was detected at all

Virus^a^	IFV	HAdV	HBoV	RSV	HCoV	HPIV	HMPV	RV
Cases with virus in MEF	1 (50.0%)	3 (14.3%)	0 (0%)	10 (40.0%)	13 (48.1%)	3 (42.9%)	4 (33.3%)	38 (48.7%)
Cases with virus in NPS	1 (50.0%)	18 (85.7%)	0 (0%)	15 (60.0%)	14 (51.9%)	4 (57.1%)	8 (66.7%)	40 (51.3%)
Concordance rate	0/2 (0%)	2/19 (10.5%)	0/0 (0%)	6/19 (31.6%)	5/22 (22.7%)	1/6 (16.7%)	3/9 (33.3%)	24/54 (44.4%)

^a^ Viral abbreviations: *IFV* Influenza virus, *HAdV* Adenovirus, *HBoV* Human bocavirus, *RSV* Respiratory syncytial virus, *HCoV* Human coronavirus, *HPIV* Parainfluenza virus, *HMPV* Human metapneumovirus, *RV* Rhinovirus

## Discussion

The microbiome of the middle ear in children with rAOM has remained relatively unexplored. Modest proportions of children with AOM carry no detectable otopathogen in the MEF by culture [5, 7] or PCR [5, 8], so there is an opportunity for the identification of novel otopathogens by studying the microbiome of the otitis-prone middle ear. Additionally, studies of the microbiome can help to identify protective bacteria that give rise to probiotic therapies [28–30], which present a novel treatment option for rAOM. Our study explored both of these opportunities and found evidence of bacterial genera that may represent novel otopathogens as well as genera that are worth further investigation as probiotic candidates. In doing so, we have, to our knowledge, characterised for the first time the microbiome of both the middle ear and nasopharynx in children with rAOM.

Our study determined that the nasopharyngeal microbiome of rAOM-resistant children is distinct from that of rAOM-prone children. Specifically, we identified three bacteria that were more abundant in our healthy controls: *Corynebacterium* (OTU8), *Dolosigranulum* (OTU5) and, to a lesser extent, *Moraxella* (OTU10). The exact *Corynebacterium* species could not be determined, but the genus *Dolosigranulum* contains only one species, *D. pigrum,* and the *Moraxella* OTU was most likely *M. lincolnii* by BLASTN. This *Moraxella* OTU was distinct from the dominant *Moraxella* (OTU2) found abundantly in the nasopharynx of both cases and controls, but OTU10 was not prevalent in the nasopharynx of all controls. The results of our study are supported by previous reports that *Corynebacterium* and *Dolosigranulum* are found in the nasopharynx of healthy adults [63] and children [64, 65] and may be associated with a decreased risk of AOM [66]. We also observed a positive correlation between *Corynebacterium* and *Dolosigranulum* which has been previously reported [67]. It has been suggested that the production of lactic acid by *Dolosigranulum* makes the surrounding environment more habitable for *Corynebacterium* species [68], which would explain the co-occurrence we observed in our study. Some studies have observed a decrease in the abundance of these genera in the nasopharynx of children who had taken antibiotics in the preceding months [65, 66], and they were also detected more frequently in the nasopharynx of breastfed compared to formula-fed 6 week old infants [69]. Together these results indicate that *Corynebacterium* and *Dolosigranulum* may be part of the normal flora of the nasopharynx, which might be strengthened by breastfeeding and disrupted by antibiotic use. In particular, *D. pigrum* is susceptible to amoxicillin [70], the recommended antibiotic for treatment of AOM. Our cases had a shorter median breastfeeding duration and higher recent antibiotic use than the controls, suggesting that maintenance of this normal flora may be important in preventing rAOM.

Regarding the potential for *Corynebacterium* and *Dolosigranulum* to actively protect against the development of rAOM in the nasopharynx of controls, protective bacteria are often closely related to the pathogens they inhibit [18, 30]. *Corynebacterium* is a member of the same family as *Turicella* (one species; *T. otitidis*) and *D. pigrum*’s closest relative is *Alloiococcus* (one species; *A. otitidis*) [71]. *Alloiococcus* and *Turicella* have both been identified as potential otopathogens [9, 72] and both were abundant in the MEF, MER and ECS of cases in this study, although they were not commonly found in the nasopharynx of our cases. Currently, the pathogenic role of *Alloiococcus* and *Turicella*, if any, is yet to be determined. Other studies have identified bacteria with a protective role that are not closely related to the pathogens they inhibit [28, 29]. In the anterior nares, *Dolosigranulum* has been linked to decreased rates of colonisation with *S. aureus* [73] and *Corynebacterium* has demonstrated activity against *S. pneumoniae* in vitro [74], indicating the potential for pathogen inhibition by these bacteria in vivo. While we have shown that *Corynebacterium* and *Dolosigranulum* are characteristic of rAOM-resistant children, determining whether they play a role in protecting against rAOM is more challenging. We controlled for antibiotic usage within the month prior to sampling, however we did not have information relevant to long-term or repeat antibiotic usage, which is common in children with rAOM. We therefore cannot exclude the possibility that *Corynebacterium* and *Dolosigranulum* are depleted in the cases due to long-term or repeated antibiotic use. Additionally, as this is a cross-sectional study we are not able to determine whether decreased *Corynebacterium* and *Dolosigranulum* precedes the development of rAOM, or if their depletion is a result of the disease. While our study is unable to answer these questions, the abundance of these genera in the controls warrants further investigation to determine whether they have a protective role in the nasopharynx.

As well as identifying potentially protective bacterial species we also investigated the potential for novel otopathogen identification. It is generally accepted that the otopathogens originate from the nasopharynx and ascend the Eustachian tube to cause infections in the middle ear. We found that the middle ear and nasopharynx of children with rAOM were not highly concordant, supporting similar findings from previous reports [75, 76]. *Haemophilus* was the most abundant otopathogen genus in the MEF, which corresponds with surveillance studies reporting a predominance of non-typeable *Haemophilus influenzae* (NTHi) in children with rAOM since the introduction of the pneumococcal vaccine targeting *S. pneumoniae* [5]. However, *Haemophilus, Moraxella* and *Streptococcus* were observed at low abundance in the MEF and MER compared to *Alloiococcus* (OTU1)*, Staphylococcus* (OTU3) and *Turicella* (OTU7)*,* with *Alloiococcus* the overall most abundant genus in the MEF, MER and ECS. There are many species of *Staphylococcus,* and we couldn’t identify OTU3 at species level by BLASTN. *S. aureus* has previously been isolated from the MEF of children with AOM [77] but is usually associated with chronic suppurative OM where the tympanic membrane is perforated [78, 79]. The other genera each contain only one species (*A. otitidis* and *T. otitidis* respectively) which have previously been identified as possible otopathogens [9, 72]. At present, the role of these bacteria in OM is still under debate [10, 80]. *A. otitidis* has been frequently detected [8, 81–83] at high abundance [81, 82] in MEF, usually from children with OME; but *T. otitidis* has been less well studied. Recently, its abundance was associated with older children (> 24 months) with COME [84], though we did not observe this pattern in our cohort. Unlike the known otopathogens, *Alloiococcus, Staphylococcus* and *Turicella* have been identified as members of the normal ear canal flora in both children and adults [85] and in our study were more abundant in the ECS than in the MEF. Also unlike the otopathogens, *Turicella* was almost absent from the nasopharynx of our cases, whilst *Alloiococcus* and *Staphylococcus* were also uncommon in this niche. *Alloiococcus* has previously been isolated infrequently from the nasopharynx of children with upper respiratory tract infections or a history of AOM [8, 10]. The rarity of *Alloiococcus* and *Turicella* in the nasopharynx suggests that they may typically colonise the middle ear. Few studies have investigated the normal flora of the healthy middle ear; when healthy, this site contains no fluid and is inaccessible without a surgical procedure. Those that have sampled this site in healthy children did not report *Alloiococcus* [86, 87] and *Turicella* appears to have only been observed once in the healthy middle ear of an adult [87]. While *Alloiococcus, Staphylococcus* and *Turicella* seem to have the potential to be novel otopathogens, we cannot yet exclude the possibility that they are normal aural flora.

We also observed increased abundance of *Alloiococcus, Staphylococcus* and *Turicella* in the MER compared to the MEF; whilst *Haemophilus* was more common in the MEF and *Streptococcus* and *Moraxella* were not significantly different between the two sample types. It is possible that *Alloiococcus, Staphylococcus* and *Turicella* adhere to the mucosa and were more efficiently sampled by the MER; differences between the middle ear and NPS were more pronounced when comparing the MER than MEF, suggesting the MER might better represent the microbiome of the middle ear. Alternatively, the MER may include contamination from the ear canal, as *Alloiococcus, Staphylococcus* and *Turicella* were the most dominant organisms in the canal and were significantly more abundant here than in the MEF. We observed increased diversity in the MER compared to the MEF, though our differential abundance analysis revealed significant differences only between genera that were common in both sites. The increased diversity may therefore include low abundance contaminant OTUs, suggesting that sampling methods to obtain the MER may be more prone to environmental or skin contamination than the MEF. However, as there were no genera unique to the ear canal and the genera at this location were found abundantly in both the ear canal and middle ear it is difficult to determine whether the canal flora contaminates the middle ear fluid with *Alloiococcus, Staphylococcus* and *Turicella* during sampling.

We observed that the nasopharyngeal microbiome of the cases was significantly more diverse than that of controls. This is in contrast to studies of the gut microbiome (a high-diversity environment [88]), where greater diversity has been associated with a healthy state, and a decrease in diversity is characteristic of disease [89, 90]. However, studies of the vaginal microbiome (a low-diversity environment [91]) have shown a similar pattern to that observed in the nasopharynx in our study; that a lower diversity is observed in the healthy environment with an increased diversity being characteristic of disease [92, 93]. Previous studies suggest that the nasopharynx is dominated by only a few taxa [64, 65] so it may be that this pattern is characteristic of less complex microbiomes. Additionally, a study in the adult nasopharynx determined that a more diverse microbiome was more susceptible to colonisation with *S. pneumoniae* [63]*.* We cannot determine from our cross-sectional study whether the higher diversity in the cases occurs before or after the development of rAOM, or as a consequence of repeat antibiotic treatment where the normal nasopharyngeal microbiome is disrupted, perhaps allowing the community to diversify. A study in adults has indicated that the gut microbiome can begin to resemble its original state in as little as one week after antibiotic treatment [94], though this recovery is often incomplete. The microbiome of adults with cystic fibrosis is similarly resilient to short-term antibiotics [95]. However, antibiotic-induced changes in the microbiome in children may be more long-term. In the gut microbiome of children, reduced richness can persist for up to 2 years after the use of macrolides [96]. Similarly, after long-term oral and inhaled antibiotic use the taxonomic richness of the microbiome in children with cystic fibrosis is markedly reduced [97]. It is possible that in our cohort, the nasopharyngeal microbiome of rAOM-prone children has been altered by antibiotic use that extends beyond the month prior to sampling that we controlled for. However, based on these studies, a reduction in microbial diversity would be expected with repeated antibiotic use; this is the opposite effect to what we observed in our cases.

The increased diversity in the case nasopharynx was contributed to in part by the presence of *Gemella* (OTU12) and *Neisseria* (OTU13), which positively correlated with each other. The OTU representative sequences matched multiple species above 97% identity with BLASTN, however the SILVA database classified OTU13 as *N. lactamica,* which is a commensal of the nasopharynx in children [98]. While *Gemella* and *Neisseria* were characteristic of the nasopharynx in children with rAOM, their abundance in the middle ear was significantly lower, suggesting these genera are not involved in pathogenesis in the middle ear and are unlikely to be novel otopathogens. Both *Neisseria* and *Gemella* have been observed in the nasopharynx of children with upper respiratory illness and AOM [66], though *Neisseria* has also previously been reported amongst the healthy flora of the nasopharynx [64, 99]. It is therefore unclear whether these genera play a role in the nasopharynx of children with rAOM or if they represent a shift in the composition of the microbiome due to repeated antibiotic usage or other factors.

Contrary to the results of our study, Hilty et al. [99] and Pettigrew et al. [66] both reported decreased diversity in the nasopharynx of children with AOM compared to children without AOM. The nasopharyngeal samples in these studies were taken during an episode of AOM, so possibly better represent the nasopharyngeal microbiome at this time. However, the majority of healthy controls used for comparison did not attend day care and most of the cases did not have a history of recurrent AOM (or this information was not provided), so these cohorts may represent less extreme ends of the phenotypic spectrum of disease.

Episodes of AOM often occur after a viral upper respiratory tract infection, [100] with bocavirus (HBoV) [101], rhinovirus (RV) [6] and respiratory syncytial virus (RSV) [102] commonly found in children with AOM. Rhinovirus was the most frequently detected virus in the nasopharynx of both our cases and controls. We detected other viruses more frequently in the cases than controls, though the odds were not always significant as relatively few viruses were detected overall. The odds of detecting respiratory syncytial virus were significantly higher in the cases than controls, which supports its association with an increased risk of AOM [103]. We also observed human metapneumovirus (HMPV) in the nasopharynx of a small number of cases, but it was absent from all of the controls, suggesting an association with rAOM. HMPV has previously been observed in 5% of children with upper respiratory tract infections, with half of these also experiencing AOM [104]. Our results were similar to the patterns of viral detection in the nasopharynx in an independent Western Australian cohort of children with and without rAOM [6], though the overall rate of viral detection was higher in that study (rAOM: 94% with at least one virus detected, controls: 71%, compared to our 61% and 48% respectively). This may be because they additionally tested for polyomavirus and enterovirus, which they found in 36% and 17% of children with rAOM respectively; for polyomavirus this was significantly higher than in their healthy controls. Bocavirus was not detected in any of the cases or controls in our study, although it has previously been seen in children with rAOM [6, 101]. We detected viruses less often in the middle ear than in the nasopharynx, which Wiertsema et al. [6] also reported. Despite lower viral identification rates, results from our cohort therefore seem to follow the same pattern as previously reported in Western Australian children.

Rhinovirus [105] and respiratory syncytial virus [106] have been observed to enhance the adherence of *S. pneumoniae* to epithelial cells in vitro. It has previously been reported that rhinovirus presence correlates with presence by standard culture of each of the three major otopathogens [107], and adenovirus with *M. catarrhalis* presence assessed by culture and PCR [6, 107]*.* We could not observe this in our study as 16S rRNA gene sequencing could not identify the otopathogen species; sequences that are more than 97% identical are grouped into the one OTU, which we could only identify at genus level. It is possible that *Haemophilus, Streptococcus* and *Moraxella* OTUs include both the otopathogens and commensal species from the same genus, which would obscure viral/otopathogen correlations.

One limitation specific to our study is that grommet surgeries are generally not performed during active infection, so the microbiome of our rAOM-prone children may not be fully representative of the microbiome during an episode of AOM. 16S rRNA studies in general can provide a comprehensive overview of the taxonomic composition of the microbiome, but are limited in that they do not provide information on microbiome function or gene content. Additionally, there are important biases to consider when conducting these studies. DNA extraction methods [108] and amplicon primers [39] work with variable efficiency across bacterial taxa, which can result in the underrepresentation of some bacteria. Our DNA extraction protocol and amplicon primers were chosen based on recommendations by Yuan et al. [108] and Klindworth et al. [39] respectively to reduce this bias. The number of copies of the 16S rRNA gene can vary amongst bacteria [109], even between strains [110] which can inflate OTU abundance. Additionally, copies within a single genome are not always identical [111], which can inflate the number of OTUs detected. Samples can be contaminated with DNA found in reagents and the laboratory environment [112], and there is the potential in our study for contamination during sample collection (i.e. from the ear canal or anterior nares). Contamination can heavily influence low biomass environments [112, 113], however we sequenced positive and negative controls to address this (see Additional file 4). Furthermore, it is difficult to achieve species-level identification with 16S rRNA sequencing as related species are often very similar in this region and the efficiency of classification also varies depending on the region of the 16S rRNA gene [114]. There is the possibility that there are commensal bacteria that we could not detect which are within the same OTU as the otopathogens. For example, *Haemophilus haemolyticus* and *Streptococcus salivarius* are closely related to otopathogens and have shown promise in other studies as candidates for the prevention of rAOM [21, 115]. Metagenomic shotgun sequencing addresses the issue of species-level identification as it sequences across the entire genome, and can thereby also provide information on gene content and function. However, DNA sequencing itself can only detect bacterial presence and does not indicate bacterial viability or activity. Metatranscriptomics addresses this issue, but this is a relatively new field and has not yet been applied to OM. The precautions taken in our study aimed to reduce the biases inherent to 16S rRNA sequencing, however we acknowledge that the relative abundance of taxa may not reflect the true proportions of bacteria and the genera we have detected may contain multiple species which are not necessarily viable or active.

## Conclusions

Our study has provided the first comprehensive exploration of the microbiome of the middle ear and nasopharynx in children with rAOM. We have taken an important step in the identification of candidate therapeutic bacteria derived from the healthy microbiome by observing significantly higher proportions of *Corynebacterium* and *Dolosigranulum* in the nasopharynx of healthy controls. Further research should focus on investigating their potential to inhibit the known otopathogens, and it would be of interest for longitudinal studies to determine whether the abundance of these genera change prior to or as a result of rAOM. We have also identified *Alloiococcus, Staphylococcus* and *Turicella* as potential otopathogens, though their relative absence in the nasopharynx and abundance in the ear canal means we cannot rule out their role as normal aural flora. *Gemella* and *Neisseria* contribute to increased diversity in the nasopharynx of children with rAOM, but do not appear to colonise the middle ear and are therefore not likely to be novel otopathogens. Shotgun metagenomics and metatranscriptomics are the next steps towards achieving species-level or strain-level resolution of these bacteria of interest and confirming their viability and investigating their activity in the middle ear. Our study has contributed significantly towards greater understanding of the microbiome of the upper respiratory tract in children with rAOM, and has taken an important step towards the development of specific probiotic therapies for the disease.

## Additional files

Additional file 1: Questionnaire completed by families recruited to the study. (PDF 28 kb)

Additional file 2: **Figure S1.** Diagrammatic overview of the 16S rRNA gene data analysis pipeline. Names of the software or tools used are in red. The SILVA database replaced the default taxonomy database in QIIME (GreenGenes) as GreenGenes 13_8 version does not discriminate between *Alloiococcus* and *Dolosigranulum.* (PDF 366 kb)

Additional file 3: **Table S1.** Full taxonomy of all OTUs identified in this study. Taxonomy is from the SILVA database, v123 for QIIME. Taxonomy assigned to OTUs by UCLUST v1.2.22q within QIIME v1.9.1 (assign_taxonomy.py). (XLSX 13 kb)

Additional file 4: Results from the positive and negative sequencing controls, including Table S2. (DOCX 27 kb)

Additional file 5: **Figure S2.** Procrustes analysis of raw and rarefied datasets. The rarefied dataset was subsampled at a threshold of 1499 reads per sample. The raw dataset excluded samples below this depth. *P*-values are non-parametric and are based on 999 Monte Carlo simulations. (PNG 174 kb)

Additional file 6: **Figure S3.** Beta diversity PCoA in the nasopharynx of cases and controls, sorted by other covariates. Case and control nasopharyngeal samples shown in Fig. 3 are coloured by other covariates. NA refers to samples where the covariate was not applicable or was missing (not given or recorded “unknown”) and the number represents individual samples. (PDF 564 kb)

Additional file 7: **Table S3.** Complete list of significantly differentially abundant OTUs determined by metagenomeSeq. All differentially abundant OTUs between a) MEF and MEF; b) MEF and NPS; c) MER and NPS; d) ECS and MEF are shown with their log fold change, *p*-values and mean and median abundance. OTUs in bold/grey are those above the selected threshold of at least 0.35% mean or median abundance in at least one group, and were present in both sets of samples where applicable. Set 1 refers to the set of samples were the left or right ear was chosen at random; these numbers are reported in results. Set 2 refers to the set of samples where the opposite ear was chosen; these results were only used for validation of the differentially abundant OTUs from Set 1. (XLSX 29 kb)

Additional file 8: **Figure S4.** Beta diversity PCoA in the samples from the cases, sorted by other covariates. Samples from the cases shown in Fig. 5 are coloured by other covariates. NA refers to samples where the covariate was not applicable or was missing (not given or recorded “unknown”) and the number represents individual samples (multiple samples per child). (PDF 392 kb)

Additional file 9: **Figure S5.** Procrustes analysis of left and right ear samples. The dataset includes both MEF and MER samples in left/right ear pairs from the same child. Samples with less than 1499 reads are excluded. The *p*-value is non-parametric and is based on 999 Monte Carlo simulations. (PNG 91 kb)

Additional file 10: **Figure S6.** Procrustes analysis of MEF and MER samples. The dataset includes pairs of MEF and MER samples from the same ear of the same child. Samples with less than 1499 reads are excluded. The *p*-value is non-parametric and is based on 999 Monte Carlo simulations. (PNG 80 kb)

Additional file 11: **Table S4.** All correlations between OTUs determined by SparCC. Correlations within each sample type are listed in separate sheets. This includes correlations between all possible pairs of OTUs found in the samples. P-values are non-parametric and were calculated as the proportion of times a correlation coefficient more extreme than the observed correlation coefficient occurred in 100 simulated datasets. (XLSX 443 kb)

## Abbreviations

AOM: Acute otitis media
CSS: Cumulative-sum-scale
DNA: Deoxyribonucleic acid
ECS: Ear canal swab
ENT: Ear, nose and throat
HAdV: Human adenovirus
HBoV: Human bocavirus
HCoV: Human coronavirus
HMPV: Human metapneumovirus
HPIV: Human parainfluenza virus
HREC: Human research ethics committee
IFV: Influenza virus
IS: Inverse Simpson
MEF: Middle ear fluid
MER: Middle ear rinse
NPS: Nasopharyngeal swab
OM: Otitis media
OME: Otitis media with effusion
OTU: Operational taxonomic unit
PCoA: Principal coordinates analysis
PCR: Polymerase chain reaction
PD: Phylogenetic diversity
rAOM: Recurrent acute otitis media
rRNA: Ribosomal ribonucleic acid
RSV: Respiratory syncytial virus
RV: Rhinovirus
STGGB: Skim milk tryptone glucose glycerol broth
TE: Tris-ethylenediaminetetraacetic acid

## References

[1] Kong K, Coates HLC (2009). Natural history, definitions, risk factors and burden of otitis media. Med J Aust.

[2] Teele DW, Klein JO, Rosner B, Greater Boston Otitis Media Study Group (1989). Epidemiology of Otitis media during the first seven years of life in children in greater Boston: a prospective, cohort study. J Infect Dis.

[3] Taylor PS, Faeth I, Marks MK, Del Mar CB, Skull SA, Pezzullo ML (2009). Cost of treating otitis media in Australia. Expert Rev Pharmacoecon Outcomes Res.

[4] Thornton RB, Rigby PJ, Wiertsema SP, Filion P, Langlands J, Coates HL (2011). Multi-species bacterial biofilm and intracellular infection in otitis media. BMC Pediatr.

[5] Wiertsema SP, Kirkham L-AS, Corscadden KJ, Mowe EN, Bowman JM, Jacoby P (2011). Predominance of nontypeable Haemophilus influenzae in children with otitis media following introduction of a 3 + 0 pneumococcal conjugate vaccine schedule. Vaccine.

[6] Wiertsema SP, Chidlow GR, Kirkham L-AS, Corscadden KJ, Mowe EN, Vijayasekaran S (2011). High detection rates of nucleic acids of a wide range of respiratory viruses in the nasopharynx and the middle ear of children with a history of recurrent acute otitis media. J Med Virol.

[7] Aguilar L, Alvarado O, Soley C, Abdelnour A, Dagan R, Arguedas A (2009). Microbiology of the middle ear fluid in Costa Rican children between 2002 and 2007. Int J Pediatr Otorhinolaryngol.

[8] Kaur R, Adlowitz DG, Casey JR, Zeng M, Pichichero ME (2010). Simultaneous assay for four bacterial species including Alloiococcus otitidis using multiplex-PCR in children with culture negative acute Otitis media. Pediatr Infect Dis J.

[9] von Graevenitz A, Funke G (2014). Turicella otitidis and Corynebacterium Auris: 20 years on. Infection.

[10] Tano K, Von Essen R, Eriksson P-O, Sjöstedt A (2008). Alloiococcus otitidis—otitis media pathogen or normal bacterial flora?. APMIS.

[11] Venekamp RP, Sanders SL, Glasziou PP, Del Mar CB, Rovers MM. Antibiotics for acute otitis media in children. Cochrane Database Syst Rev. 2015;(6). Art. No.: CD000219. 10.1002/14651858.CD000219.pub4 PMC7043305 26099233

[12] Klein JO (2003). Bacterial resistance and antimicrobial drug selection. Evidence based Otitis media.

[13] Boston M, McCook J, Burke B, Derkay C (2003). Incidence of and risk factors for additional tympanostomy tube insertion in children. Arch Otolaryngol Head Neck Surg.

[14] FAO/WHO. Guidelines for the evaluation of probiotics in food. 2002. http://www.who.int/foodsafety/fs_management/en/probiotic_guidelines.pdf. Accessed 31 May 2017.

[15] Bermudez-Brito M, Plaza-Díaz J, Muñoz-Quezada S, Gómez-Llorente C, Gil A (2012). Probiotic mechanisms of action. Ann Nutr Metab.

[16] Rautava S, Salminen S, Isolauri E (2008). Specific probiotics in reducing the risk of acute infections in infancy – a randomised, double-blind, placebo-controlled study. Br J Nutr.

[17] Stecksén-Blicks C, Sjöström I, Twetman S (2009). Effect of long-term consumption of milk supplemented with Probiotic lactobacilli and fluoride on dental caries and general health in preschool children: a cluster-randomized study. Caries Res.

[18] Di Pierro F, Donato G, Fomia F, Adami T, Careddu D, Cassandro C (2012). Preliminary pediatric clinical evaluation of the oral probiotic streptococcus salivarius K12 in preventing recurrent pharyngitis and/or tonsillitis caused by streptococcus pyogenes and recurrent acute otitis media. Int J Gen Med.

[19] Roos K, Hakansson EG, Holm S (2001). Effect of recolonisation with “interfering” (alpha) streptococci on recurrences of acute and secretory otitis media in children: randomised placebo controlled trial. Br Med J Int Ed.

[20] Skovbjerg S, Roos K, Holm SE, Håkansson EG, Nowrouzian F, Ivarsson M (2009). Spray bacteriotherapy decreases middle ear fluid in children with secretory otitis media. Arch Dis Child.

[21] Marchisio P, Santagati M, Scillato M, Baggi E, Fattizzo M, Rosazza C (2015). Streptococcus salivarius 24SMB administered by nasal spray for the prevention of acute otitis media in otitis-prone children. Eur J Clin Microbiol Infect Dis.

[22] Hatakka K, Savilahti E, Pönkä A, Meurman JH, Poussa T, Näse L (2001). Effect of long term consumption of probiotic milk on infections in children attending day care centres: double blind, randomised trial. BMJ.

[23] Hatakka K, Blomgren K, Pohjavuori S, Kaijalainen T, Poussa T, Leinonen M (2007). Treatment of acute otitis media with probiotics in otitis-prone children—a double-blind, placebo-controlled randomised study. Clin Nutr.

[24] Taipale T, Pienihäkkinen K, Isolauri E, Larsen C, Brockmann E, Alanen P (2011). Bifidobacterium animalis subsp. lactis BB-12 in reducing the risk of infections in infancy. Br J Nutr.

[25] Taipale TJ, Pienihäkkinen K, Isolauri E, Jokela JT, Söderling EM (2016). Bifidobacterium animalis subsp. lactis BB-12 in reducing the risk of infections in early childhood. Pediatr Res.

[26] Cohen R, Martin E, La Rocque F de, Thollot F, Pecquet SM, Werner A, et al. Probiotics and Prebiotics in preventing episodes of acute Otitis Media in High-Risk Children: a randomized, double-blind, Placebo-controlled Study ET J 2013;32:810–814. 10.1097/INF.0b013e31828df4f3 23429555

[27] Tano K, Grahn Håkansson E, Holm SE, Hellström S (2002). A nasal spray with alpha-haemolytic streptococci as long term prophylaxis against recurrent otitis media. Int J Pediatr Otorhinolaryngol.

[28] Lawley TD, Clare S, Walker AW, Stares MD, Connor TR, Raisen C (2012). Targeted restoration of the intestinal microbiota with a simple, defined Bacteriotherapy resolves relapsing Clostridium Difficile disease in mice. PLoS Pathog.

[29] Casey PG, Gardiner GE, Casey G, Bradshaw B, Lawlor PG, Lynch PB (2007). A five-strain probiotic combination reduces pathogen shedding and alleviates disease signs in pigs challenged with salmonella enterica Serovar Typhimurium. Appl Environ Microbiol.

[30] Iwase T, Uehara Y, Shinji H, Tajima A, Seo H, Takada K (2010). Staphylococcus Epidermidis Esp inhibits Staphylococcus Aureus biofilm formation and nasal colonization. Nature.

[31] Brennan-Jones CG, Whitehouse AJ, Park J, Hegarty M, Jacques A, Eikelboom RH (2015). Prevalence and risk factors for parent-reported recurrent otitis media during early childhood in the western Australian pregnancy cohort (Raine) study. J Paediatr Child Health.

[32] Paradise JL, Rockette HE, Colborn DK, Bernard BS, Smith CG, Kurs-Lasky M (1997). Otitis media in 2253 Pittsburgh-area infants: prevalence and risk factors during the first two years of life. Pediatrics.

[33] Teo SM, Mok D, Pham K, Kusel M, Serralha M, Troy N (2015). The infant airway microbiome in health and disease impacts later asthma development. Cell Host Microbe.

[34] Chidlow GR, Harnett GB, Shellam GR, Smith DW (2009). An economical tandem multiplex real-time PCR technique for the detection of a comprehensive range of respiratory pathogens. Viruses.

[35] Lee W-M, Kiesner C, Pappas T, Lee I, Grindle K, Jartti T (2007). A diverse group of previously unrecognized human rhinoviruses are common causes of respiratory illnesses in infants. PLoS One.

[36] Stephens DS, Swartley JS, Kathariou S, Morse SA (1991). Insertion of Tn916 in Neisseria meningitidis resulting in loss of group B capsular polysaccharide. Infect Immun.

[37] Quast C, Pruesse E, Yilmaz P, Gerken J, Schweer T, Yarza P (2013). The SILVA ribosomal RNA gene database project: improved data processing and web-based tools. Nucleic Acids Res.

[38] Yilmaz P, Parfrey LW, Yarza P, Gerken J, Pruesse E, Quast C (2014). The SILVA and “all-species living tree project (LTP)” taxonomic frameworks. Nucleic Acids Res.

[39] Klindworth A, Pruesse E, Schweer T, Peplies J, Quast C, Horn M (2013). Evaluation of general 16S ribosomal RNA gene PCR primers for classical and next-generation sequencing-based diversity studies. Nucleic Acids Res.

[40] Andrews S (2010). FastQC: a quality control tool for high throughput sequence data.

[41] Edgar RC (2013). UPARSE: highly accurate OTU sequences from microbial amplicon reads. Nat Methods.

[42] Edgar RC (2010). Search and clustering orders of magnitude faster than BLAST. Bioinformatics.

[43] Caporaso JG, Kuczynski J, Stombaugh J, Bittinger K, Bushman FD, Costello EK (2010). QIIME allows analysis of high-throughput community sequencing data. Nat Methods.

[44] Walters T. strip_primers_fastq.py. 2015. https://gist.github.com/walterst/2c592044b3b9e44a4290. Accessed 20 Apr 2017.

[45] Schmieder R, Edwards R (2011). Fast identification and removal of sequence contamination from genomic and metagenomic datasets. PLoS One.

[46] Caporaso JG, Bittinger K, Bushman FD, DeSantis TZ, Andersen GL, Knight R (2010). PyNAST: a flexible tool for aligning sequences to a template alignment. Bioinforma Oxf Engl.

[47] DeSantis TZ, Hugenholtz P, Larsen N, Rojas M, Brodie EL, Keller K (2006). Greengenes, a chimera-checked 16S rRNA gene database and workbench compatible with ARB. Appl Environ Microbiol.

[48] Price MN, Dehal PS, Arkin AP (2010). FastTree 2 – approximately maximum-likelihood trees for large alignments. PLoS One.

[49] McMurdie PJ, Holmes S (2014). Waste Not. Want Not: Why Rarefying Microbiome Data Is Inadmissible PLOS Comput Biol.

[50] R Core Team. R: A language and environment for statistical computing. Vienna, Austria: R Foundation for Statistical Computing; 2013. http://www.R-project.org/. Accessed 20 Apr 2017.

[51] Fay MP (2010). Confidence intervals that match Fisher’s exact or Blaker’s exact tests. Biostat Oxf Engl.

[52] Faith DP (1992). Conservation evaluation and phylogenetic diversity. Biol Conserv.

[53] Simpson E (1949). Measurement of Diversity Nature.

[54] Wickham H (2009). ggplot2: elegant graphics for data analysis.

[55] Auguie B. gridExtra: Miscellaneous Functions for “Grid” Graphics. 2016. https://CRAN.R-project.org/package=gridExtra. Accessed 20 Apr 2017.

[56] Lozupone C, Knight R (2005). UniFrac: a new phylogenetic method for comparing microbial communities. Appl Environ Microbiol.

[57] Vázquez-Baeza Y, Pirrung M, Gonzalez A, Knight R (2013). EMPeror: a tool for visualizing high-throughput microbial community data. GigaScience.

[58] Friedman J, Alm EJ (2012). Inferring correlation networks from genomic survey data. PLoS Comput Biol.

[59] Paulson JN, Talukder H, Pop M, Bravo HC. metagenomeSeq: Statistical analysis for sparse high-throughput sequencing. Bioconductor package: 1.18.0. http://cbcb.umd.edu/software/metagenomeSeq. Accessed 26 Oct 2017.

[60] Paulson JN, Stine OC, Bravo HC, Pop M (2013). Differential abundance analysis for microbial marker-gene surveys. Nat Methods.

[61] Barter R, Yu B. Superheat: a graphical tool for exploring complex datasets using Heatmaps. R package version 0.1.0. https://arxiv.org/abs/1512.01524.

[62] Zhang Z, Schwartz S, Wagner L, Miller W (2000). A greedy algorithm for aligning DNA sequences. J Comput Biol.

[63] Cremers AJ, Zomer AL, Gritzfeld JF, Ferwerda G, van Hijum SA, Ferreira DM (2014). The adult nasopharyngeal microbiome as a determinant of pneumococcal acquisition. Microbiome..

[64] Bogaert D, Keijser B, Huse S, Rossen J, Veenhoven R, van Gils E (2011). Variability and diversity of nasopharyngeal microbiota in children: a metagenomic analysis. PLoS One.

[65] Biesbroek G, Wang X, Keijser BJF, Eijkemans RMJ, Trzciński K, Rots NY (2014). Seven-Valent pneumococcal conjugate vaccine and nasopharyngeal microbiota in healthy children. Emerg Infect Dis.

[66] Pettigrew MM, Laufer AS, Gent JF, Kong Y, Fennie KP, Metlay JP (2012). Upper respiratory tract microbial communities, acute Otitis media pathogens, and antibiotic use in healthy and sick children. Appl Environ Microbiol.

[67] Laufer AS, Metlay JP, Gent JF, Fennie KP, Kong Y (2011). Pettigrew MM. Microbial Communities of the Upper Respiratory Tract and Otitis Media in Children mBio.

[68] de Steenhuijsen Piters WAA, Sanders EAM, Bogaert D (2015). The role of the local microbial ecosystem in respiratory health and disease. Phil Trans R Soc B.

[69] Biesbroek G, Bosch AATM, Wang X, Keijser BJF, Veenhoven RH, Sanders EAM (2014). The impact of breastfeeding on nasopharyngeal microbial communities in infants. Am J Respir Crit Care Med.

[70] Laclaire L, Facklam R (2000). Antimicrobial susceptibility and clinical sources of Dolosigranulum pigrum cultures. Antimicrob Agents Chemother.

[71] Vos P, Garrity G, Jones D, Krieg NR, Ludwig W, Rainey FA, et al. Bergey’s manual of systematic bacteriology: volume 3: the Firmicutes. New York: Springer Science & Business Media; 2009.

[72] Hendolin PH, Kärkkäinen U, Himi T, Markkanen A, Ylikoski J (1999). High incidence of Alloiococcus otitis in otitis media with effusion. Pediatr Infect Dis J.

[73] Liu CM, Price LB, Hungate BA, Abraham AG, Larsen LA, Christensen K (2015). Staphylococcus Aureus and the ecology of the nasal microbiome. Sci Adv.

[74] Bomar L, Brugger SD, Yost BH, Davies SS, Lemon KP (2016). Corynebacterium accolens releases Antipneumococcal free fatty acids from human nostril and skin surface Triacylglycerols. MBio.

[75] Kaur R, Czup K, Casey JR, Pichichero ME (2014). Correlation of nasopharyngeal cultures prior to and at onset of acute otitis media with middle ear fluid cultures. BMC Infect Dis.

[76] van Dongen TMA, van der Heijden GJMG, van Zon A, Bogaert D, Sanders EAM, Schilder AGM (2013). Evaluation of concordance between the microorganisms detected in the nasopharynx and middle ear of children with otitis media. Pediatr Infect Dis J.

[77] Veenhoven R, Bogaert D, Uiterwaal C, Brouwer C, Kiezebrink H, Bruin J (2003). Effect of conjugate pneumococcal vaccine followed by polysaccharide pneumococcal vaccine on recurrent acute otitis media: a randomised study. Lancet.

[78] Choi HG, Park KH, Park SN, Jun BC, Lee DH, Yeo SW (2010). The appropriate medical management of methicillin-resistant Staphylococcus Aureus in chronic suppurative otitis media. Acta Otolaryngol (Stockh).

[79] Jang CH, Chang-Hun S, Wang P-C (2004). Topical vancomycin for chronic suppurative otitis media with methicillin-resistant Staphylococcus Aureus otorrhoea. J Laryngol Otol Devon.

[80] Holzmann D, Funke G, Linder T, Nadal D (2002). Turicella otitidis and Corynebacterium Auris do not cause otitis media with effusion in children. Pediatr Infect Dis J.

[81] Jervis-Bardy J, Rogers GB, Morris PS, Smith-Vaughan HC, Nosworthy E, Leong LEX (2015). The microbiome of otitis media with effusion in indigenous Australian children. Int J Pediatr Otorhinolaryngol.

[82] Chan CL, Wabnitz D, Bardy JJ, Bassiouni A, Wormald P-J, Vreugde S (2016). The microbiome of otitis media with effusion. Laryngoscope.

[83] Ashhurst-Smith C, Hall ST, Walker P, Stuart J, Hansbro PM, Blackwell CC (2007). Isolation of Alloiococcus otitidis from indigenous and non-indigenous Australian children with chronic otitis media with effusion. FEMS Immunol Med Microbiol.

[84] Krueger A, Val S, Pérez-losada M, Panchapakesan K, Devaney J, Duah V (2017). Relationship of the middle ear effusion microbiome to Secretory Mucin production in pediatric patients with chronic Otitis media. Pediatr Infect Dis J.

[85] Stroman DW, Roland PS, Dohar J, Burt W (2001). Microbiology of normal external Auditory Canal. Laryngoscope.

[86] Westerberg BD, Kozak FK, Thomas EE, Blondel-Hill E, Brunstein JD, Patrick DM (2009). Is the healthy middle ear a normally sterile site?. Otol Neurotol.

[87] Minami SB, Mutai H, Suzuki T, Horii A, Oishi N, Wasano K, et al. Microbiomes of the normal middle ear and ears with chronic otitis media. Laryngoscope. 2017; 10.1002/lary.26579. 10.1002/lary.26579 28397271

[88] Gill SR, Pop M, DeBoy RT, Eckburg PB, Turnbaugh PJ, Samuel BS (2006). Metagenomic analysis of the human distal gut microbiome. Science.

[89] Chang JY, Antonopoulos DA, Kalra A, Tonelli A, Khalife WT, Schmidt TM (2008). Decreased diversity of the fecal microbiome in recurrent Clostridium Difficile-associated diarrhea. J Infect Dis.

[90] Manichanh C, Rigottier-Gois L, Bonnaud E, Gloux K, Pelletier E, Frangeul L (2006). Reduced diversity of faecal microbiota in Crohn’s disease revealed by a metagenomic approach. Gut.

[91] The Human Microbiome Project Consortium (2012). Structure, function and diversity of the healthy human microbiome. Nature.

[92] Oakley BB, Fiedler TL, Marrazzo JM, Fredricks DN (2008). Diversity of human vaginal bacterial communities and associations with clinically defined bacterial Vaginosis. Appl Environ Microbiol.

[93] Ling Z, Kong J, Liu F, Zhu H, Chen X, Wang Y (2010). Molecular analysis of the diversity of vaginal microbiota associated with bacterial vaginosis. BMC Genomics.

[94] Dethlefsen L, Relman DA (2011). Incomplete recovery and individualized responses of the human distal gut microbiota to repeated antibiotic perturbation. Proc Natl Acad Sci U S A.

[95] Fodor AA, Klem ER, Gilpin DF, Elborn JS, Boucher RC, Tunney MM (2012). The adult cystic fibrosis airway microbiota is stable over time and infection type, and highly resilient to antibiotic treatment of exacerbations. PLoS One.

[96] Korpela K, Salonen A, Virta LJ, Kekkonen RA, Forslund K, Bork P (2016). Intestinal microbiome is related to lifetime antibiotic use in Finnish pre-school children. Nat Commun.

[97] Klepac-Ceraj V, Lemon KP, Martin TR, Allgaier M, Kembel SW, Knapp AA (2010). Relationship between cystic fibrosis respiratory tract bacterial communities and age, genotype, antibiotics and Pseudomonas Aeruginosa. Environ Microbiol.

[98] Gold R, Goldschneider I, Lepow ML, Draper TF, Randolph M (1978). Carriage of Neisseria meningitidis and Neisseria lactamica in infants and children. J Infect Dis.

[99] Hilty M, Qi W, Brugger SD, Frei L, Agyeman P, Frey PM (2012). Nasopharyngeal microbiota in infants with acute Otitis media. J Infect Dis.

[100] Chonmaitree T, Revai K, Grady JJ, Clos A, Patel JA, Nair S (2008). Viral upper respiratory tract infection and otitis media complication in young children. Clin Infect Dis.

[101] Lehtoranta L, Söderlund-Venermo M, Nokso-Koivisto J, Toivola H, Blomgren K, Hatakka K (2012). Human bocavirus in the nasopharynx of otitis-prone children. Int J Pediatr Otorhinolaryngol.

[102] Heikkinen T, Thint M, Chonmaitree T (1999). Prevalence of various respiratory viruses in the middle ear during acute otitis media. N Engl J Med.

[103] Sagai S, Suetake M, Yano H, Yoshida M, Ohyama K, Endo H (2004). Relationship between respiratory syncytial virus infection and acute otitis media in children. Auris Nasus Larynx.

[104] Williams JV, Wang CK, Yang C-F, Tollefson SJ, Heck JM, House FS (2006). The role of human Metapneumovirus in upper respiratory tract infections in children: a 20-year experience. J Infect Dis.

[105] Ishizuka S, Yamaya M, Suzuki T, Takahashi H, Ida S, Sasaki T (2003). Effects of rhinovirus infection on the adherence of Streptococcus Pneumoniae to cultured human airway epithelial cells. J Infect Dis.

[106] Hament J-M, Aerts PC, Fleer A, van Dijk H, Harmsen T, Kimpen JLL (2004). Enhanced adherence of Streptococcus Pneumoniae to human epithelial cells infected with respiratory Syncytial virus. Pediatr Res.

[107] Jacoby P, Watson K, Bowman J, Taylor A, Riley TV, Smith DW (2007). Modelling the co-occurrence of Streptococcus Pneumoniae with other bacterial and viral pathogens in the upper respiratory tract. Vaccine.

[108] Yuan S, Cohen DB, Ravel J, Abdo Z, Forney LJ (2012). Evaluation of methods for the extraction and purification of DNA from the human microbiome. PLoS One.

[109] Větrovský T, Baldrian P (2013). The variability of the 16S rRNA gene in bacterial genomes and its consequences for bacterial community analyses. PLoS One.

[110] Greenblum S, Carr R, Borenstein E (2015). Extensive strain-level copy-number variation across human gut microbiome species. Cell.

[111] Pei AY, Oberdorf WE, Nossa CW, Agarwal A, Chokshi P, Gerz EA (2010). Diversity of 16S rRNA genes within individual prokaryotic genomes. Appl Environ Microbiol.

[112] Salter SJ, Cox MJ, Turek EM, Calus ST, Cookson WO, Moffatt MF (2014). Reagent and laboratory contamination can critically impact sequence-based microbiome analyses. BMC Biol.

[113] Lauder AP, Roche AM, Sherrill-Mix S, Bailey A, Laughlin AL, Bittinger K (2016). Comparison of placenta samples with contamination controls does not provide evidence for a distinct placenta microbiota. Microbiome.

[114] Chakravorty S, Helb D, Burday M, Connell N, Alland D (2007). A detailed analysis of 16S ribosomal RNA gene segments for the diagnosis of pathogenic bacteria. J Microbiol Methods.

[115] Pickering JL, Prosser A, Corscadden KJ, de Gier C, Richmond PC, Zhang G, et al. Haemophilus haemolyticus interaction with host cells is different to Nontypeable Haemophilus influenzae and prevents NTHi association with epithelial cells. Front Cell Infect Microbiol. 2016;6 10.3389/fcimb.2016.00050. 10.3389/fcimb.2016.00050 PMC4860508 27242968

