# Supplementary material for: A microbiome case-control study of recurrent acute otitis media identified potentially protective bacterial genera
Source: BMC Microbiol. 2018 Feb 20;18:13. doi: 10.1186/s12866-018-1154-3 (PMC5819196; doi:10.1186/s12866-018-1154-3)
Supplement: Supplementary file 1 — Questionnaire completed by families recruited to the study. (PDF 28 kb) [file 12866_2018_1154_MOESM1_ESM.pdf]

Defining the microbes in the middle ear and upper respiratory tract that lead to recurrent ear infections

Data Collection Form

History

Child's DOB __ __ / __ __ / __ __ **I.D. Number: M G O __ __ __ __**

Gender: ☐ Male ☐ Female

Identify with being Aboriginal or Torres Straight Islander? ☐ Yes ☐ No

Suburb (where participant lives)**:** ______________________________ Postcode:________

Who is providing the information? ☐Mother ☐Father ☐Grandparent ☐Guardian ☐Foster Carer ☐Other________________

Is the child currently breastfed? ☐ Yes ☐ No ☐N/A ☐ Unknown

If Yes: ☐ Exclusive ☐ Partial ☐ Unknown

How old was child when breastfeeding ceased? __________________ ☐ Unknown

Is the child attending day care or school? ☐ Day care ☐ School ☐ Neither ☐N/A ☐ Unknown

If at day care/school, how many hours/wk _________

What age (in months) did they start day care_________

Number of people normally living in your house

Total number __ __ Number of children (at or under 5 years of age) __ __

Does anyone at home smoke? ☐ Yes ☐ No ☐ Unknown

If yes: How many people? __ __ ☐ Unknown

If yes: How often do they smoke? ☐ Occasionally ☐ Regularly ☐ Unknown

If yes: Does anyone smoke inside your house? ☐ Inside ☐ Outside only ☐ Unknown

Does your child have any chronic illnesses such as: If Yes, describe

Ear infection ☐ Yes ☐ No ☐ Unknown ______________________________

If yes, How many ear infections have they had in total? ___________________ ☐ Unknown

Has your child previously had grommets inserted? ☐ Yes ☐ No ☐ Unknown

Has your child previously had their adenoids removed? ☐ Yes ☐ No ☐ Unknown

Asthma ☐ Yes ☐ No ☐ Unknown ______________________________

Allergy ☐ Yes ☐ No ☐ Unknown ______________________________

Chest problem ☐ Yes ☐ No ☐ Unknown ______________________________

Heart problem ☐ Yes ☐ No ☐ Unknown ______________________________

Kidney problem ☐ Yes ☐ No ☐ Unknown ______________________________

Any other illnesses ☐ Yes ☐ No ☐ Unknown ______________________________

Has your child ever been admitted to hospital for infection? ☐ Yes ☐ No ☐ Unknown

Please specify (Hospital, date and site - LRTI, meningitis, gastrointestinal, UTI etc)

____

____

____

Antibiotic usage during the last month: ☐Yes ☐ No ☐ Unknown

If yes: Date started__ __/ __ __/ __ __ Date finished__ __/ __ __/ __ __

Name of Antibiotic given:

☐ Penicillin ☐ Cephalexin ☐ Erythromycin

☐ Amoxycillin ☐ Cefaclor ☐ Roxithromycin

☐ Augmentin ☐ Ceftriaxone ☐ Azithromycin

☐ Flucloxacillin ☐ Cotrimoxazole ☐ Other____________

GP Name: ____

Practice Name: ____

Address: ____

____

Examination

General health (on day of surgery)

Nose

Any Nasal discharge? ☐ Yes ☐ No ☐ Unknown

If Yes, Discharge severity: ☐ Mild ☐ Moderate ☐ Profuse ☐ Unknown

Discharge Colour: ☐ Clear ☐ Opaque ☐ Yellow/green ☐ Unknown

Ear

Any ear discharge? ☐ Yes ☐ No ☐ Unknown

If yes, which ear? ☐ Left ☐ Right ☐ Both ☐ Unknown

General

Has the child been diagnosed with..

Cleft lip/palate? ☐ Yes ☐ No ☐ Unknown

Immune deficiency? ☐ Yes ☐ No ☐ Unknown

Any genetic syndrome? ☐ Yes ☐ No ☐ Unknown

Immunisation Status

Your child's current immunisation status will be checked on the Australian Childhood Immunisation Register

ACIR checked by (researcher name): ____________________________________

Date ACIR checked __ __ / __ __/____

Name of Research Staff: _____________________________ **Signature**:__________________________
(Printed)

Date of interview __ __ / __ __/____
