# Supplementary figures and images for: A microbiome case-control study of recurrent acute otitis media identified potentially protective bacterial genera
Source: BMC Microbiol. 2018 Feb 20;18:13. doi: 10.1186/s12866-018-1154-3 (PMC5819196; doi:10.1186/s12866-018-1154-3)

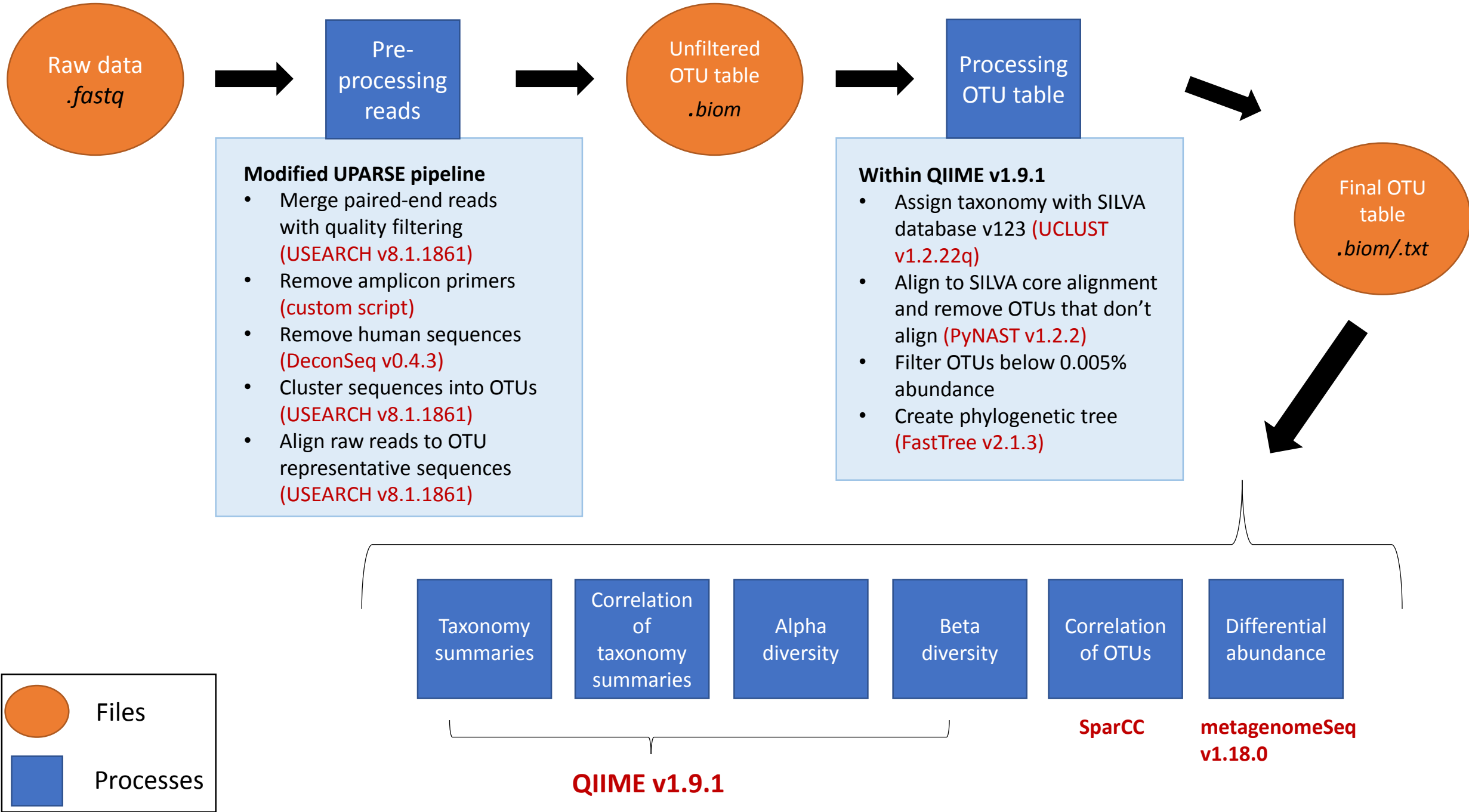

Supplement: Supplementary file 2 — Figure S1. Diagrammatic overview of the 16S rRNA gene data analysis pipeline. Names of the software or tools used are in red. The SILVA database replaced the default taxonomy database in QIIME (GreenGenes) as GreenGenes 13_8 version does not discriminate between Alloiococcus and Dolosigranulum. (PDF 366 kb) [file 12866_2018_1154_MOESM2_ESM.pdf]

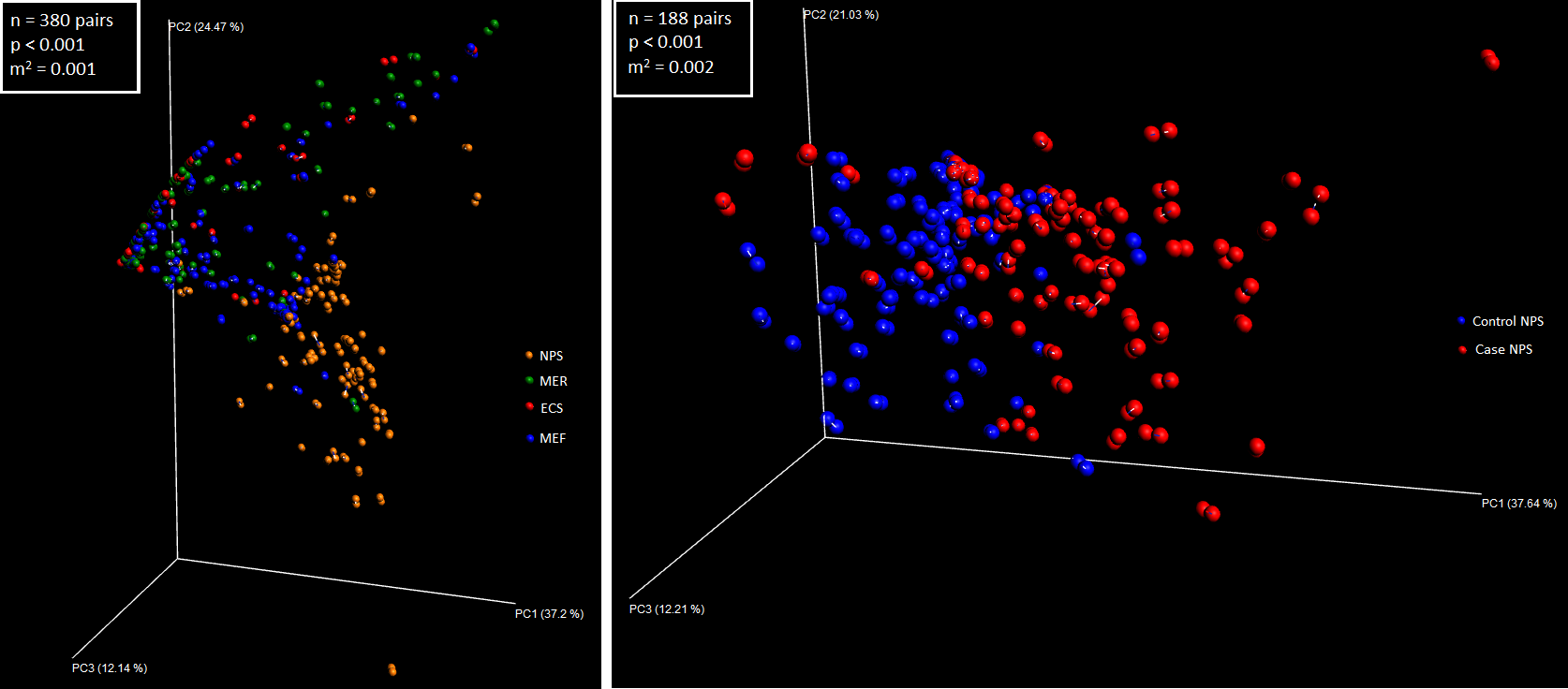

Supplement: Supplementary file 5 — Figure S2. Procrustes analysis of raw and rarefied datasets. The rarefied dataset was subsampled at a threshold of 1499 reads per sample. The raw dataset excluded samples below this depth. P-values are non-parametric and are based on 999 Monte Carlo simulations. (PNG 174 kb) [file 12866_2018_1154_MOESM5_ESM.png]

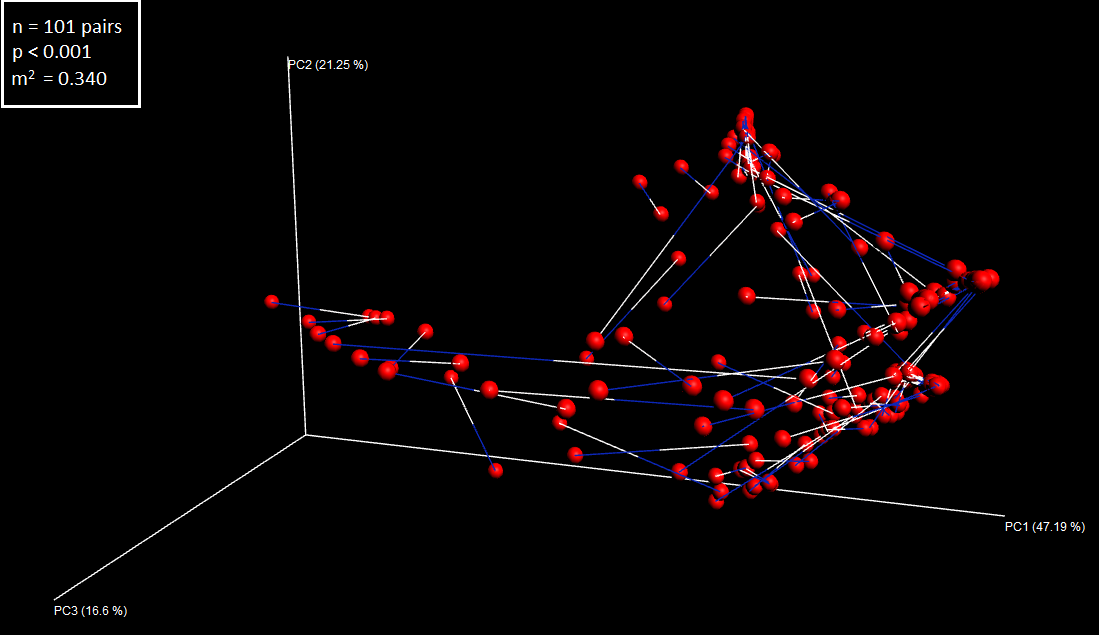

Supplement: Supplementary file 9 — Figure S5. Procrustes analysis of left and right ear samples. The dataset includes both MEF and MER samples in left/right ear pairs from the same child. Samples with less than 1499 reads are excluded. The p-value is non-parametric and is based on 999 Monte Carlo simulations. (PNG 91 kb) [file 12866_2018_1154_MOESM9_ESM.png]

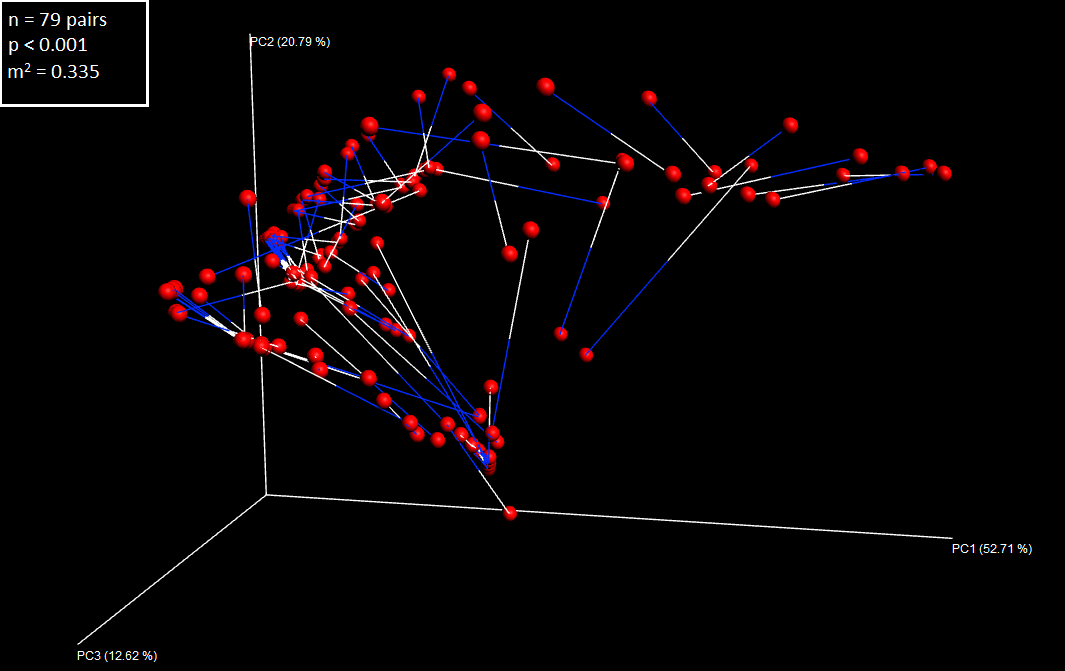

Supplement: Supplementary file 10 — Figure S6. Procrustes analysis of MEF and MER samples. The dataset includes pairs of MEF and MER samples from the same ear of the same child. Samples with less than 1499 reads are excluded. The p-value is non-parametric and is based on 999 Monte Carlo simulations. (PNG 80 kb) [file 12866_2018_1154_MOESM10_ESM.png]
