# Supplementary material for: A microbiome case-control study of recurrent acute otitis media identified potentially protective bacterial genera
Source: BMC Microbiol. 2018 Feb 20;18:13. doi: 10.1186/s12866-018-1154-3 (PMC5819196; doi:10.1186/s12866-018-1154-3)
Supplement: Supplementary file 4 — Results from the positive and negative sequencing controls, including Table S2. (DOCX 27 kb) [file 12866_2018_1154_MOESM4_ESM.docx]

**Sequencing controls**

Positive and negative sequencing controls are essential in a microbiome study. We included a positive mock community control to assess bias across independent sequencing runs.The negative controls were used to identify reagent or environmental contaminants during the DNA extraction process (negative extraction controls) or PCR amplification (negative PCR controls).

Table S2a) shows the summary of taxa for the positive sequencing control containing 16 bacterial species, which was included in duplicate on each of the four sequencing runs. These samples were highly correlated at genus level with the expected theoretical composition of 6.25% each species (Pearson correlation coefficient = 0.986, 95% CI 0.983 – 0.988, non-parametric p = 0.001, 999 permutations), indicating that separation of the samples onto four independent sequencing runs did not introduce bias.

The negative controls were assessed for taxa that may have an impact on the composition of the clinical samples, shown in Table S2b). These controls typically had a low sequence read count with 13/16 negative controls falling below the selected threshold of 1499 reads for removing low-depth samples from the analysis. The most abundant taxa across all negative controls included *Delftia* (20.0%)*, Pseudomonas* (14.4%)*, Alloiococcus* (13.4%) and *Lysinibacillus* (10.7%)*.* The dominant *Pseudomonas* OTU in the negative controls (OTU58) was distinct from the dominant *Pseudomonas* OTU found in abundance in some of the ear samples (OTU9, presumptively *Pseudomonas aeruginosa* by BLASTN at 100% identity). The presence of *Alloiococcus* in a few negative control samples suggests some cross-contamination from samples as it is known to be found in the middle ear and ear canal and was greatly abundant in the ear samples. *Delftia* and *Lysinibacillus* are known environmental colonisers and these are found at a very low relative abundance in the clinical samples (median <0.003% and mean <0.1% relative abundance over all samples included in analysis). Furthermore, the negative controls were moderately correlated with each other at genus level when comparing taxonomy summaries. Pairs of extraction and PCR negative controls from the same sequencing plate (one of each per plate) had a Pearson correlation coefficient of 0.598 (95% CI 0.537-0.653, non-parametric p = 0.001, 999 permutations). Pairs of negative extraction controls on the same sequencing run had a Pearson correlation coefficient of 0.507 (95% CI 0.405-0.596, non-parametric p = 0.001, 999 permutations) and pairs of negative PCR controls had a coefficient of 0.519 (95% CI 0.420-0.607, non-parametric p = 0.004, 999 permutations). This indicates that while there is a pattern amongst the negative controls, they do not contain a strong, consistent contaminant signal and are unlikely to influence the conclusions drawn from the samples due to the extremely low abundance of major contaminant taxa.

| **Genus** | **Expected** | MOCK1 | MOCK2 | MOCK3 | MOCK4 | MOCK5 | MOCK6 | MOCK3 | MOCK3 |
| --- | --- | --- | --- | --- | --- | --- | --- | --- | --- |
| *Corynebacterium* | **6.25** | 4.83 | 5.03 | 4.08 | 3.94 | 4.80 | 4.89 | 4.16 | 3.61 |
| *Propionibacterium* | **6.25** | 5.15 | 4.88 | 4.09 | 3.73 | 5.19 | 5.37 | 4.97 | 4.81 |
| *Staphylococcus* | **18.75** | 20.67 | 19.98 | 21.94 | 22.12 | 21.38 | 21.06 | 22.00 | 22.80 |
| *Globicatella* | **6.25** | 5.92 | 4.81 | 5.60 | 5.52 | 6.33 | 5.45 | 5.22 | 4.80 |
| *Alloiococcus* | **6.25** | 7.71 | 8.00 | 8.76 | 8.73 | 8.05 | 7.97 | 7.83 | 7.99 |
| *Streptococcus* | **12.50** | 12.71 | 13.64 | 11.16 | 10.83 | 12.39 | 12.57 | 12.35 | 13.02 |
| *Veillonella* | **6.25** | 6.13 | 5.95 | 5.70 | 5.56 | 5.95 | 5.50 | 6.09 | 5.76 |
| *Neisseria* | **6.25** | 6.66 | 6.60 | 7.30 | 6.83 | 6.22 | 5.70 | 7.06 | 6.59 |
| *Klebsiella* | **6.25** | 7.14 | 7.30 | 8.17 | 8.22 | 7.02 | 7.11 | 7.03 | 6.47 |
| *Haemophilus* | **12.50** | 10.39 | 10.80 | 9.71 | 11.60 | 10.27 | 11.97 | 10.35 | 11.83 |
| *Moraxella* | **6.25** | 6.68 | 6.99 | 6.92 | 6.58 | 6.28 | 6.58 | 6.82 | 6.92 |
| *Pseudomonas* | **6.25** | 6.01 | 6.00 | 6.52 | 6.31 | 6.11 | 5.83 | 6.09 | 5.34 |
| Other (47 other taxa) | **0.00** | 0.01 | 0.01 | 0.05 | 0.02 | 0.01 | 0.01 | 0.02 | 0.05 |

**Table S2:** **Taxonomic composition of the a) positive control (MOCK) replicates and b) negative extraction and PCR controls.** Numbers represent column percentages. Taxa below an average relative abundance of 1% across all replicates were collapsed into “Other”.

**a)**

| **Genus** | **Overall** | Negex1 | Negex11 | Negex13 | Negex19 | Negex32 | Negex46 | Negex55 | Negex64 | NegPCR1 | NegPCR2 | NegPCR3 | NegPCR4 | NegPCR5 | NegPCR6 | NegPCR7 | NegPCR8 |
| --- | --- | --- | --- | --- | --- | --- | --- | --- | --- | --- | --- | --- | --- | --- | --- | --- | --- |
| *Corynebacterium* | **1.12** | 0.39 | 0.00 | 0.05 | 7.29 | 0.24 | 3.98 | 1.36 | 0.00 | 0.04 | 0.00 | 0.68 | 3.29 | 0.12 | 0.26 | 0.17 | 0.00 |
| *Propionibacterium* | **1.18** | 0.19 | 0.00 | 1.57 | 2.13 | 0.00 | 13.93 | 0.78 | 0.00 | 0.00 | 0.00 | 0.02 | 0.00 | 0.00 | 0.13 | 0.17 | 0.00 |
| *Bacillus* | **8.27** | 28.90 | 0.00 | 29.46 | 16.82 | 6.93 | 0.00 | 4.08 | 12.90 | 0.04 | 12.67 | 0.00 | 0.00 | 0.84 | 19.61 | 0.00 | 0.00 |
| *Geobacillus* | **1.49** | 13.87 | 0.00 | 4.07 | 2.94 | 0.00 | 0.00 | 1.94 | 1.08 | 0.00 | 0.00 | 0.00 | 0.00 | 0.00 | 0.00 | 0.00 | 0.00 |
| *Lysinibacillus* | **10.69** | 0.00 | 26.50 | 0.00 | 9.02 | 0.00 | 44.78 | 4.08 | 0.00 | 0.00 | 0.00 | 0.00 | 12.62 | 12.76 | 0.00 | 14.14 | 47.16 |
| *Staphylococcus* | **3.64** | 4.43 | 3.18 | 0.16 | 8.61 | 0.00 | 8.96 | 0.58 | 15.05 | 0.38 | 1.73 | 3.30 | 1.18 | 1.20 | 0.65 | 5.79 | 3.01 |
| *Alloiococcus* | **13.51** | 0.00 | 18.02 | 4.61 | 14.79 | 53.65 | 7.46 | 17.28 | 20.43 | 0.50 | 0.58 | 2.34 | 60.82 | 1.93 | 6.58 | 6.30 | 0.84 |
| *Rhizobium* | **6.51** | 5.01 | 1.77 | 5.53 | 2.84 | 0.00 | 0.50 | 16.12 | 0.00 | 20.40 | 10.29 | 0.29 | 2.51 | 10.23 | 21.03 | 5.79 | 1.84 |
| *Delftia* | **19.97** | 27.55 | 27.56 | 44.87 | 2.03 | 1.22 | 0.00 | 12.23 | 0.00 | 73.39 | 72.18 | 0.00 | 0.16 | 29.36 | 0.00 | 18.91 | 10.03 |
| *Escherichia-Shigella* | **5.47** | 0.00 | 0.00 | 0.00 | 0.00 | 0.00 | 0.00 | 0.00 | 0.00 | 0.00 | 0.00 | 87.57 | 0.00 | 0.00 | 0.00 | 0.00 | 0.00 |
| *Haemophilus* | **4.21** | 9.44 | 0.00 | 2.01 | 8.00 | 3.77 | 3.98 | 0.97 | 15.05 | 1.57 | 0.91 | 0.08 | 0.31 | 19.01 | 1.16 | 1.02 | 0.00 |
| *Acinetobacter* | **1.26** | 0.00 | 0.00 | 2.17 | 11.04 | 0.85 | 0.00 | 0.00 | 0.00 | 0.00 | 0.00 | 0.00 | 0.00 | 6.14 | 0.00 | 0.00 | 0.00 |
| *Moraxella* | **3.66** | 2.89 | 0.35 | 3.74 | 3.75 | 17.40 | 2.49 | 3.88 | 12.90 | 0.81 | 0.66 | 1.44 | 2.12 | 0.96 | 3.87 | 1.36 | 0.00 |
| *Pseudomonas* | **14.52** | 0.19 | 18.02 | 0.00 | 6.89 | 2.43 | 11.44 | 32.62 | 7.53 | 0.15 | 0.00 | 3.60 | 14.03 | 16.61 | 41.81 | 44.12 | 32.94 |
| Other (45 other taxa) | **4.50** | 7.13 | 4.59 | 1.74 | 3.85 | 13.50 | 2.49 | 4.08 | 15.05 | 2.72 | 0.99 | 0.68 | 2.98 | 0.84 | 4.90 | 2.21 | 4.18 |

**b)**
