# Supplementary material for: A microbiome case-control study of recurrent acute otitis media identified potentially protective bacterial genera
Source: BMC Microbiol. 2018 Feb 20;18:13. doi: 10.1186/s12866-018-1154-3 (PMC5819196; doi:10.1186/s12866-018-1154-3)

Samples from the cases

Antibiotic use

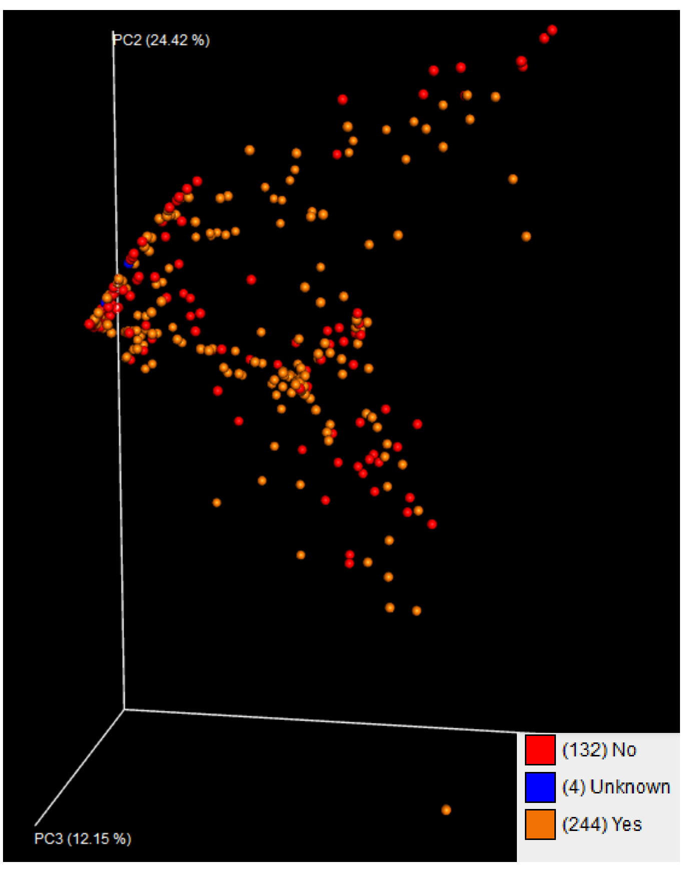

Age (increasing from white to red)

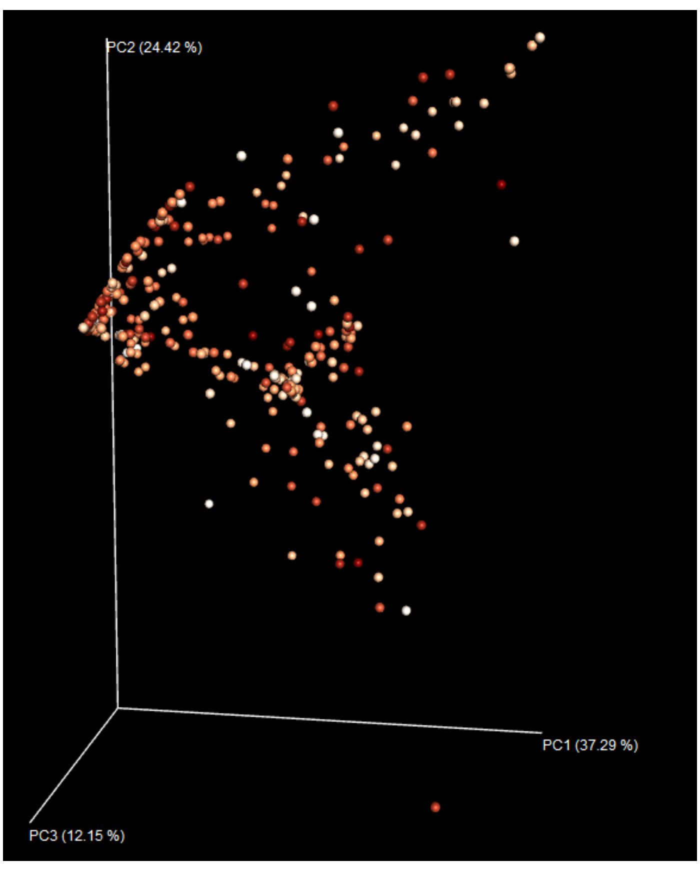

Breastfeeding duration

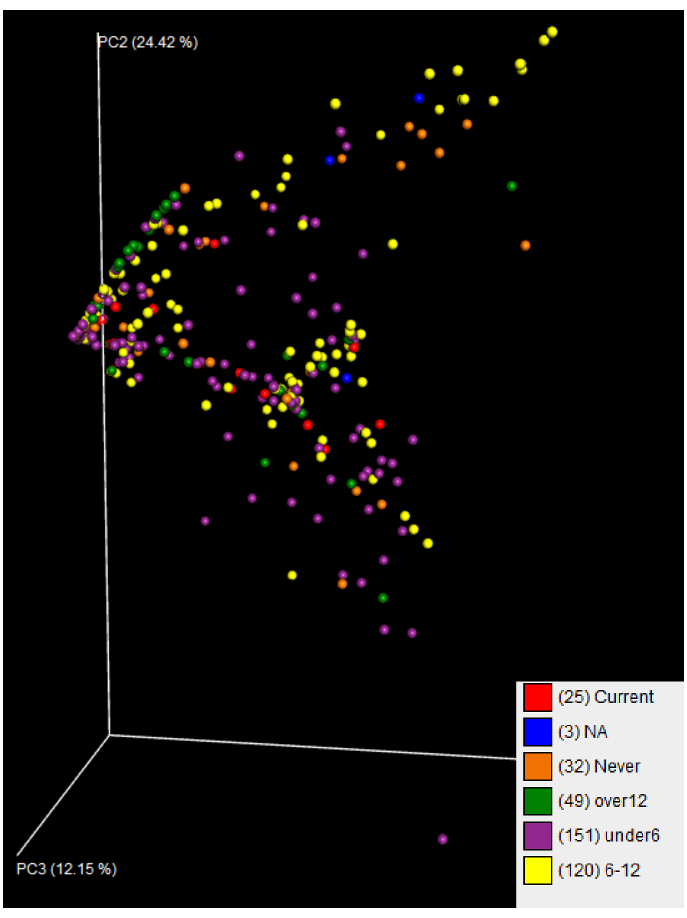

Presence of chronic illness

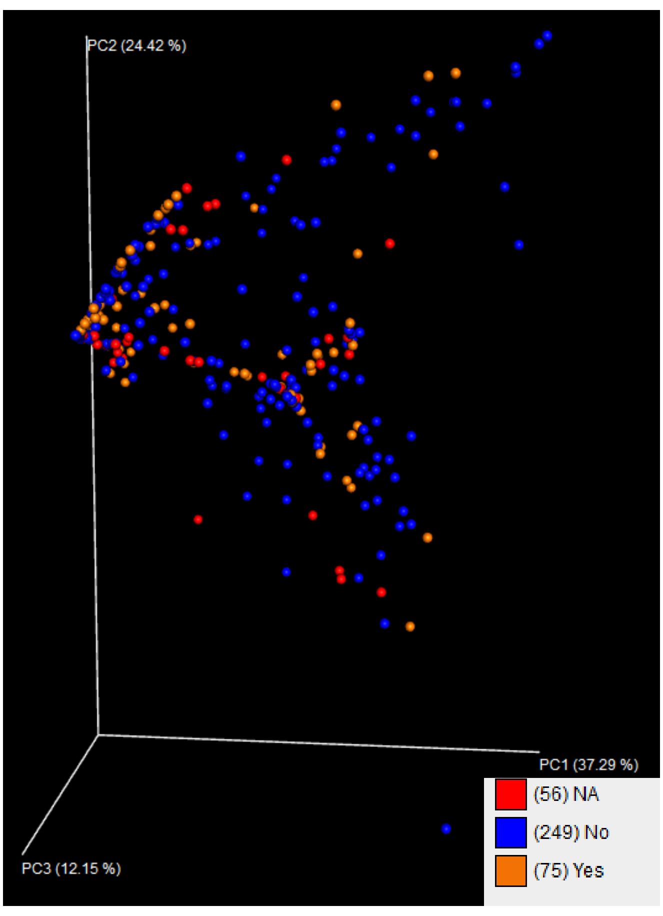

Current attendance at day care or school

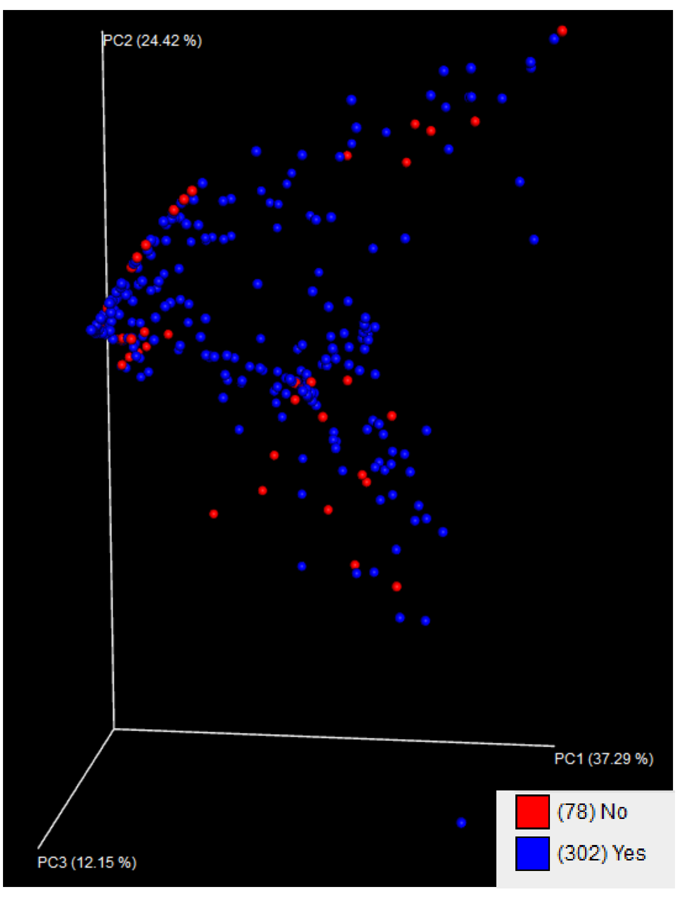

Sex

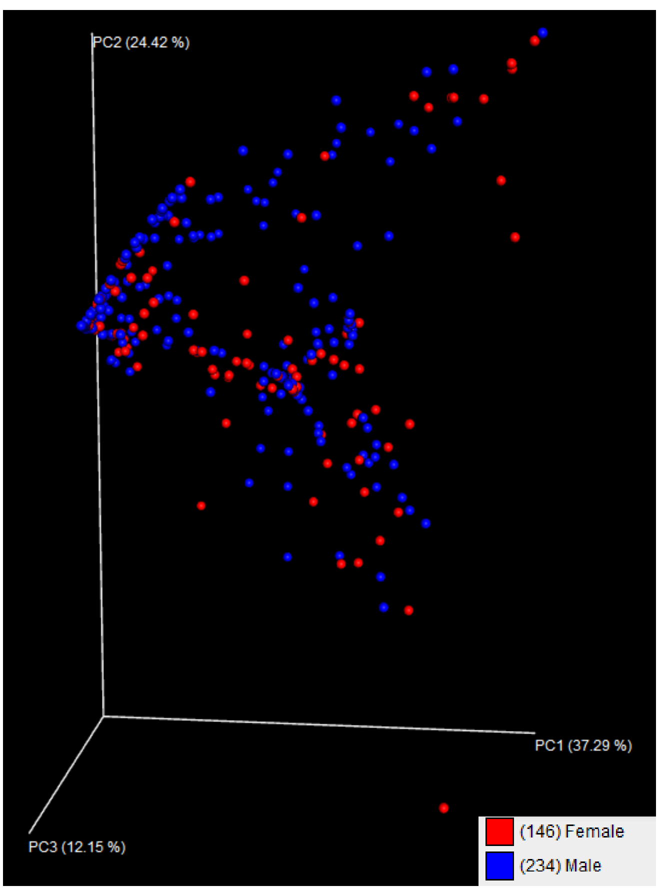

Previous admission to hospital for infection

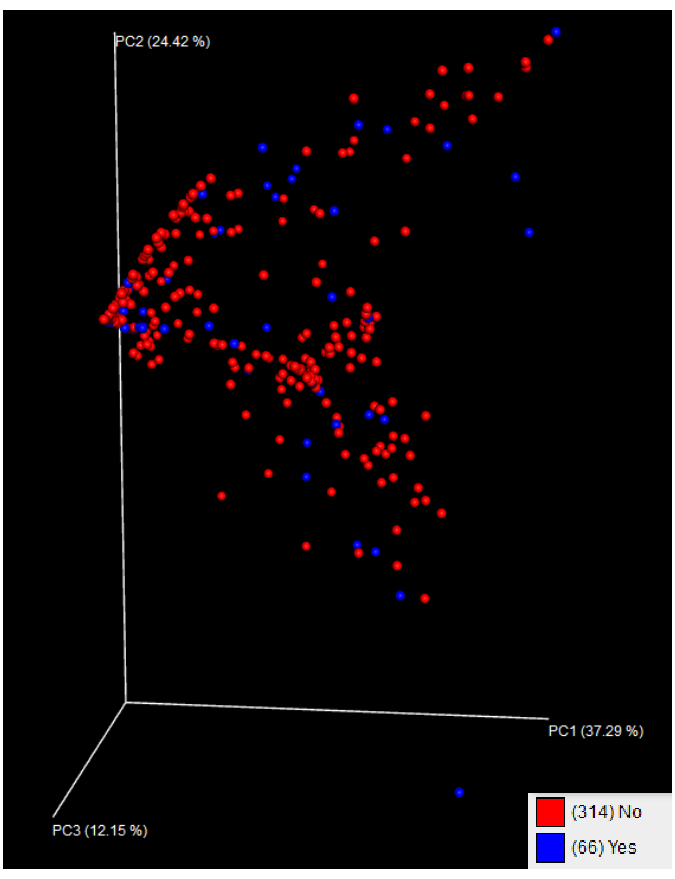

Season of collection

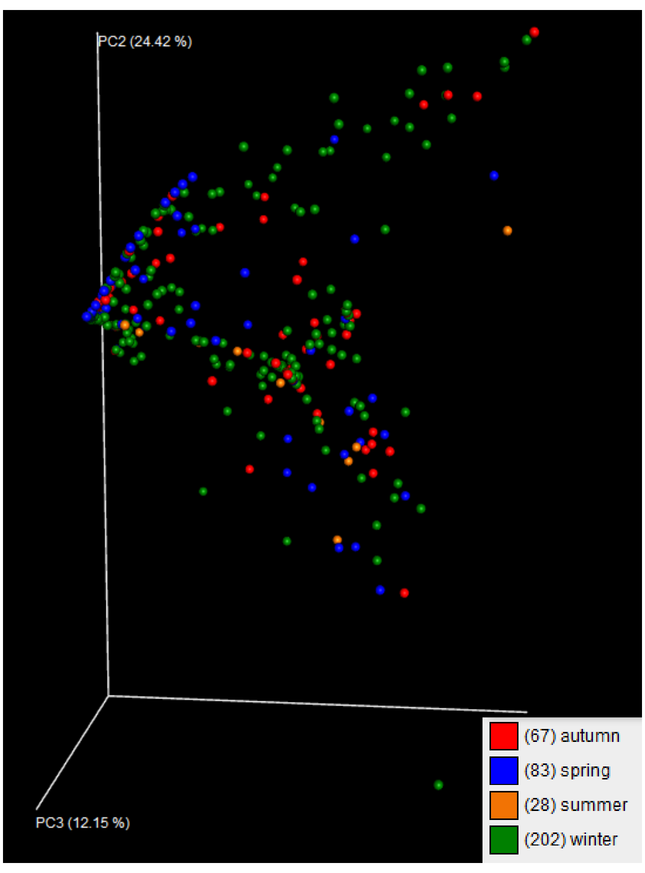

Presence of siblings in the household

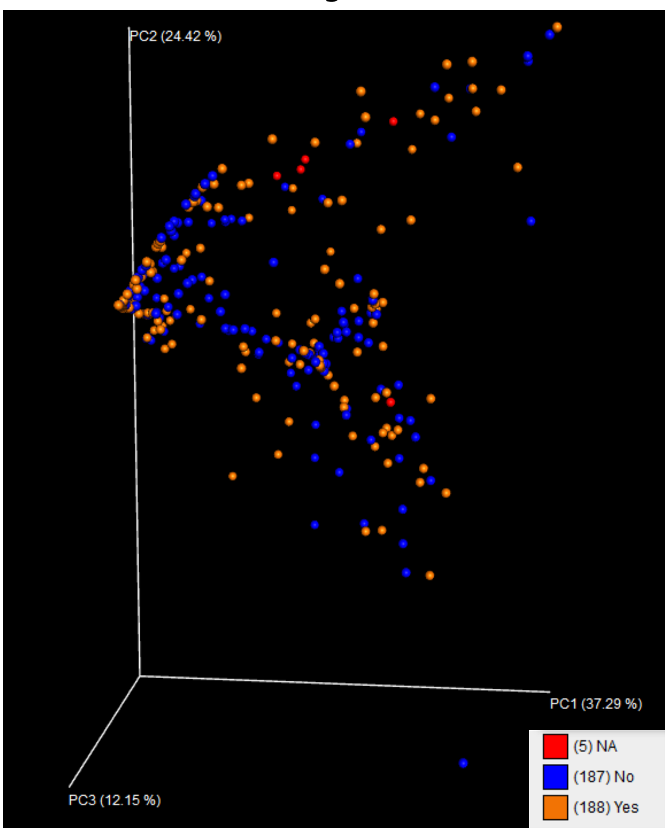

Sequencing run

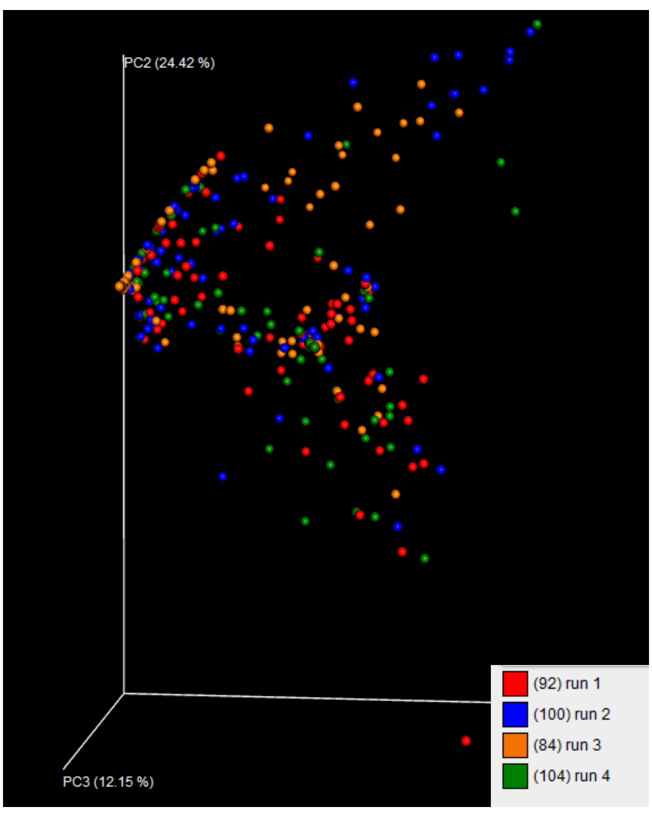

Supplement: Supplementary file 8 — Figure S4. Beta diversity PCoA in the samples from the cases, sorted by other covariates. Samples from the cases shown in Fig. 5 are coloured by other covariates. NA refers to samples where the covariate was not applicable or was missing (not given or recorded “unknown”) and the number represents individual samples (multiple samples per child). (PDF 392 kb) [file 12866_2018_1154_MOESM8_ESM.pdf]
